# Sub–10-nm imprint lithography on elastomers by chain translocated crystallization in nanochannels

**DOI:** 10.1126/sciadv.aec3829

**Published:** 2026-05-22

**Authors:** Yingchao Yang, Yunfei Ru, Shuanhu Qi, Yingzhi Sun, Jin Huang, Zhewei Yan, Tianyi Zhao, Biao Zuo, Lei Jiang, Mingjie Liu

**Affiliations:** ^1^ State Key Laboratory of Bioinspired Interfacial Materials Science, Bioinspired Science Innovation Center, Hangzhou International Innovation Institute, Beihang University, Hangzhou 311115, China.; ^2^ School of Chemistry, Beihang University, Beijing 100191, China.; ^3^ School of Physics, Beihang University, Beijing 100191, China.; ^4^ School of Chemistry and Chemical Engineering, Key Laboratory of Surface & Interface Science of Polymer Materials of Zhejiang Province, Zhejiang Sci-Tech University, Hangzhou 310018, China.; ^5^ CAS Key Laboratory of Bio-inspired Materials and Interfacial Science, Technical Institute of Physics and Chemistry Chinese Academy of Sciences, Beijing 100190, China.; ^6^ State Key Laboratory of Bioinspired Interfacial Materials Science, Suzhou Institute for Advanced Research, University of Science and Technology of China, Suzhou 215123, China.

## Abstract

Elastomers with nanostructured surfaces exhibit substantial importance in enhancing interfacial properties including mechanics and optical diffraction. However, the structure on elastomer surfaces by imprinting is limited to microscale features due to kinetically arrested reptation diffusion and inevitable entropic recovery. Herein, we design a dynamic elastomeric network to achieve nanoimprinting with ultrahigh resolution and aspect ratios by chain translocated crystallization in nanochannels. Specifically, dynamic covalent bonds trigger network reconstruction after thermal activation and enhance chain disentanglement, which promotes chain translocation in nanochannels. Meanwhile, the crystalline phase within nanochannels creates energetic barriers that effectively restrict entropy-driven recovery. This strategy enables imprinting of elastomer surfaces with sub–10-nm structures, across a 10^7^ range in length scale and aspect ratios exceeding 100:1, far outperforming conventional elastomers. Moreover, the imprinted nanostructures provide elastomer surfaces with a marked modulus enhancement by ~5 times to reach 4.2 gigapascals while simultaneously improving optical transparency, which endows elastomers with highly integrated multifunctional protective capabilities.

## Teaser

Chain translocated crystallization in nanochannels enables sub–10-nm imprint lithography on elastomers.

## INTRODUCTION

Nanostructured polymer surfaces have emerged as critical functional platforms (*1*–*3*) for advanced technologies due to their customizable interfacial properties (*4*–*7*), particularly in mechanical applications (*8*–*11*) and optical diffraction (*12*). Nanoimprint lithography represents an efficient approach for high-throughput, large-scale, and cost-effective nanofabrication with exceptional precision (*13*–*15*). Thermoplastic polymers have been widely adopted as preferred materials due to their superior processing characteristics (*13*, *16*, *17*). Now, extensive efforts have been devoted to improving nanoimprint resolution by optimizing polymer chain rheological behavior within the confined nanochannels (*18*, *19*). These strategies include molecular engineering tailoring (*20*, *21*), interfacial energy modulation (*22*), and plasticizer integration (*23*), which effectively reduce interchain friction and interface adsorption (*24*, *25*). Furthermore, the regulation of glass transition temperature via copolymerization modification enables a favorable trade-off between structural stability and flow properties (*26*, *27*). Overall, thermoplastic polymers mainly use glass transition induced by heat or light to create a dynamic processing window where chain segments have sufficient mobility to fill nanochannels and fixes the obtained structures by vitrification, which often requires a relatively long cooling time (*13*, *28*). Such advances have led to remarkable achievements in nanoimprinting, with feature size reaching 10 nm and aspect ratios of 10:1.

For elastomer systems, spontaneous shape recovery driven by network entropic elasticity after demolding substantially hinders structure fixation (*29*–*31*). Recent advances have focused on incorporating shape memory properties into elastomers to suppress this entropic recovery, thereby ensuring structural fidelity (*32*–*34*). However, the current imprinted features on elastomer surfaces remain limited to micrometer-scale dimensions. The topological constraints of cross-linked networks kinetically arrest reptation diffusion and long-range conformational relaxation of polymer chains, which restrict the accessibility of polymer chains to nanochannels and compromising the completeness of nanoconfined filling. Therefore, it is still a substantial challenge to fabricate structures with nanoscale features and high aspect ratios on elastomer surfaces.

Herein, we developed a strategy to achieve nanoimprinting on elastomer surfaces with ultrahigh resolution and aspect ratios by chain translocated crystallization in nanochannels. Dynamic covalent bonds enabled network reconstruction and enhanced chain disentanglement through bond exchange reactions, which facilitated efficient chain translocation in nanochannels. Moreover, confined crystallization that occurred at a temperature above the glass transition further restrained entropy-driven shape recovery. These crystalline phases provided higher energy barriers and restricted chain segment mobility, leading to the storage of internal stress that thermodynamically stabilized the imprinted structures. The obtained elastomer showed superior imprinting capabilities, achieving surface structures with sub–10-nm feature sizes, extending across a 10^7^ range in length scale (from tens of nanometers to millimeters) and achieving aspect ratios exceeding 100:1. On the basis of this, we developed the hierarchical impact-resistant elastomers (HIREs) with nanostructured surfaces that simultaneously enhanced modulus (up to 4.2 GPa) and optical transparency. This nanoimprinting-enabled mechanical reinforcement creates thin, elastic protective materials integrating superior impact resistance and transparency, promising for protective equipment applications.

## RESULTS

### Resolution, aspect ratio, and strategy universality

**Fig. 1.**
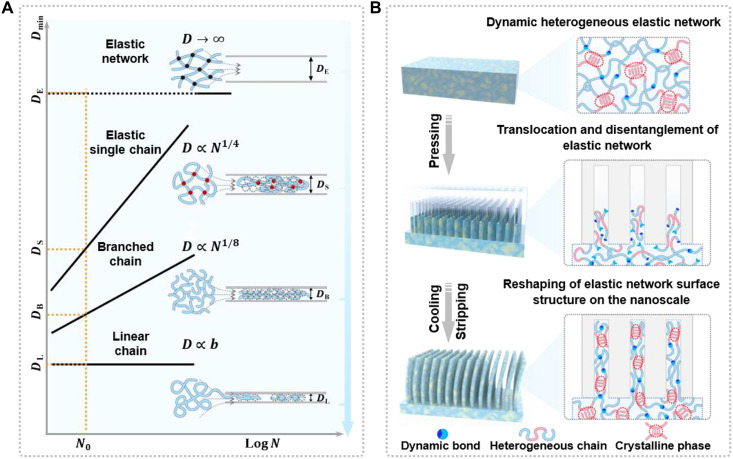
Design concept of dynamic heterogeneous elastomers for nanoimprinting by chain translocated crystallization. (**A**) Minimum diameter (*D*_min_) required for polymer chain translocation through cylindrical nanochannels with different topologies and schematic of chain translocation behaviors. (**B**) Schematic illustration showing the nanoimprint strategy and process on a cross-linked elastomeric surface.

The high-resolution and aspect ratio imprinting on elastomers faces fundamental challenges arising from kinetically arrested reptation diffusion and entropic recovery. These network characteristics severely restrict nanochannel filling and induce spontaneous shape recovery upon demolding. Theoretically, the translocation capacity of polymer chains through nanochannels strongly correlates with their topological architecture and degree of polymerization (*N*) (*35*, *36*), as reflected in the distinct minimum diameter (*D*_min_) of nanochannels required for chain passage (Fig. 1A). The minimum diameter for cross-linked elastic networks (*D*_E_) approaches infinity, indicating their inability to traverse nanochannels smaller than their bulk dimensions. In contrast, elastic single chains (*D*_S_) (individual intrachain cross-linked polymer chains) and branched chains (*D*_B_) demonstrate superior translocation capabilities with scaling relationships of $D_{S}\propto N^{\frac{1}{4}}$ and $D_{B}\propto N^{\frac{1}{8}}$, respectively. Linear chains exhibit optimal translocation behavior, dependent only on the Kuhn segment length *b* (Supplementary Text). By introducing dynamic covalent bonds, the cross-linked elastomeric network undergoes dynamic reconstruction process and enhances chain disentanglement, which promotes the chain translocation in nanochannels and achieves efficient network filling within nanochannels. The crystalline phases within nanochannels provide additional energy barriers that restrict chain segment mobility, thereby suppressing shape recovery driven by entropic rebound and ensuring structural fidelity (fig. S1 and Supplementary Text). As shown in Fig. 1B, the dynamic heterogeneous networks underwent chain translocated crystallization during imprinting, achieving the reshaping of the elastomeric network surface structure at the nanoscale. Here, we used dynamic heterogeneous gel elastomers (DHGEs) as a model system, where the network was constructed through in situ free radical copolymerization of *N*,*N*-dimethylacrylamide (DMA), stearyl methacrylate (SMA), and the disodium *N*,*N*′-diacryloyl-l-cystinate (DAC) cross-linker (fig. S2). In this case, the disulfide bonds provided by DAC enabled dynamic network reconstruction, and the phase transition of poly(stearyl methacrylate) (PSMA) promoted the formation of crystalline domains within the network. Differential scanning calorimetry (DSC) characterization revealed that the long alkyl side chains of PSMA exhibited a distinct crystallization temperature (27.8°C) and melting temperature (37.4°C) (fig. S3). When heated above the crystallization temperature, PSMA chain melting led to gel softening. At 70°C, the compressive modulus decreased to ~90 kPa. When cooled below the crystallization temperature, the recrystallization of PSMA stored the network internal stress while suppressing elastic recovery and notably increasing the compressive modulus to 6.2 MPa (fig. S4A). In addition, we investigated the influence of dynamic bonds, serving as cross-linkers, on the mechanical properties of DHGEs. A series of DHGE-*x* gels was prepared with DAC contents ranging from 2.5 to 50 wt % (where *x* represents the mass fraction of DAC relative to the DMA monomer). Mechanical testing results indicated that increasing cross-linking density simultaneously enhanced the moduli in both melted and crystallized states, without affecting the modulus difference between the two states (fig. S4B). As temperature increases, the activation of dynamic covalent bonds triggered network reconstruction, which synergistically combined with the phase transition behavior of PSMA to facilitate nanoscale surface imprinting.

**Fig. 2.**
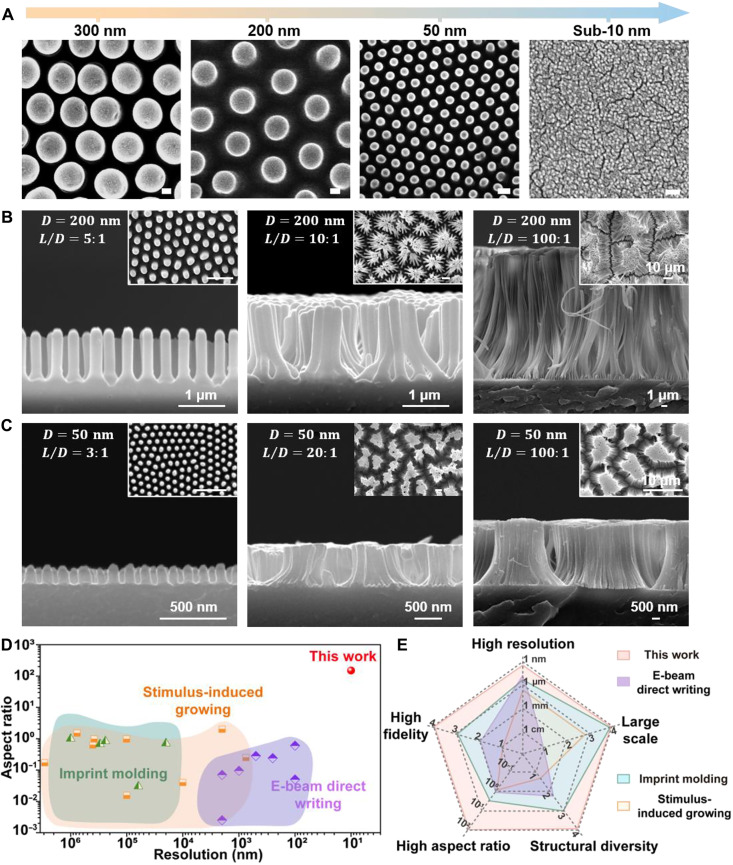
Surface imprinting characterization and performance analysis of dynamic heterogeneous elastomers. (**A**) SEM characterization of imprinting feature sizes. Scale bars, 100 nm. SEM characterization of aspect ratios at feature sizes of (**B**) 200 nm and (**C**) 50 nm. (**D**) Comparative analysis of the aspect ratio and resolution among cross-linked elastomer systems fabricated via different methods: stimulus-induced growing, imprint molding, and electron-beam direct writing. (**E**) Radar chart comparison of comprehensive performance metrics (resolution, aspect ratio, pattern fidelity, structural diversity, and scalability) between this work and conventional fabrication approaches (stimulus-induced growing, imprint molding, and electron-beam direct writing).

**Fig. 3.**
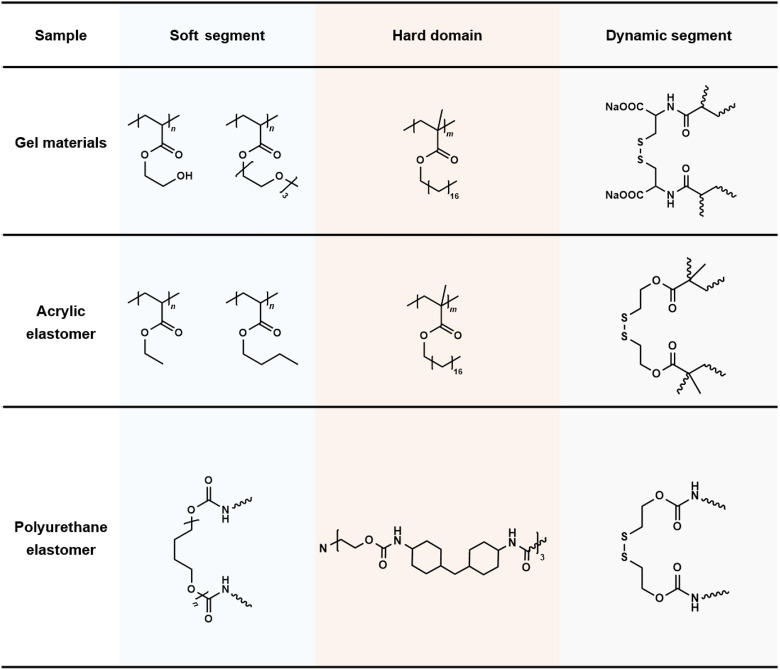
Chemical structures and network compositions of diverse elastomer systems based on the chain translocated crystallization strategy.

We systematically investigated the nanoimprint performance of DHGE materials by analyzing the feature sizes and aspect ratios through scanning electron microscopy (SEM) characterization. Unless otherwise specified, DHGE-20 was used for imprinting. For feature sizes, as shown in Fig. 2A, surface structures could be precisely imprinted from hundreds of nanometers down to sub–10 nm, covering a broad dimensional range including 300-, 200-, and 50-nm and sub–10-nm features, demonstrating ultrahigh imprint resolution (fig. S5). Additional feature sizes were demonstrated in fig. S6. To investigate the range of aspect ratios, we selected two representative diameters of 200 and 50 nm. The imprinted structures with both diameters demonstrated a wide range of aspect ratios from low (<5:1) to high (>10:1), with aspect ratios of 5:1, 10:1, and 100:1 for 200-nm structures (Fig. 2B) and 3:1, 20:1, and 100:1 for 50-nm structures (Fig. 2C and fig. S5, D and E). The insets showed top-view images of the corresponding structures. These results exhibited the excellent imprinting capabilities of DHGEs at different feature sizes, enabling the fabrication of structures with gradient aspect ratios from nanopillars to nanofibers. Notably, under high aspect ratio conditions, nanowires tended to form bundles due to capillary forces (*37*–*39*). This bundling behavior depends on material modulus, geometric parameters, and surface properties (*40*–*44*). Low-magnification SEM observations further verified that these high-fidelity nanostructures could achieve large scalable fabrication at the macroscopic scale (figs. S7 and S8). Moreover, the DHGE surface could be imprinted with diverse structures including V-shaped structures (top diameter: 100 nm, bottom diameter: 450 nm, and depth: 400 nm), circular pillars (3 μm), square pillars (5 μm), triangular patterns (50 μm), letters “BUAA” (700 μm), and periodic grooves with millimeter dimensions (fig. S9), revealing its versatility for complex patterning across length scales from tens of nanometers to millimeters and beyond.

By comparison, traditional cross-linked polymer elastomers showed limited nanoimprint resolution and aspect ratios due to their cross-linked network topology. Phase change gels (PCGs), phase separation gels (PSGs), and supramolecular gels (SPGs) with shape memory characteristics, as common functional elastomers, typically exhibited imprinting resolution at the micrometer scale with aspect ratios below 6:1. In addition, we conducted control experiments by replacing DAC with the nondynamic cross-linker ethylene glycol dimethacrylate (EGDMA). The results revealed that crystalline domains alone were insufficient to achieve such superior imprinting performance, further highlighting the crucial role of dynamic covalent reconstruction for efficient chain translocation within nanochannels (fig. S10). Furthermore, we expanded this strategy from gel materials (DHGEs) to acrylate-based elastomer [dynamic heterogeneous acrylic elastomer (DHAE)] and polyurethane-based elastomer [dynamic heterogeneous polyurethane elastomer (DHPE)] systems (Fig. 3 and figs. S11 and S12). We achieved structural imprinting on elastomer surfaces across at least seven orders of magnitude in length scale, with minimum feature sizes down to sub–10 nm and aspect ratios exceeding 100:1. These structural features far outperformed the current capabilities of nanostructures fabricated on cross-linked elastomer surfaces through stimulus-induced growth (*45*–*49*), electron-beam direct writing (*50*), and imprint molding (*32*–*34*) (Fig. 2D, table S1, and Supplementary Text). Moreover, during the demolding process, we used wet etching rather than physical demolding, which effectively prevented structural damage caused by interfacial adhesion and stress concentration, thus preserving structural integrity and achieving high fabrication yield (fig. S13). Chain translocated crystallization in nanochannels based on dynamic covalent reconstruction mechanisms notably improved the nanoimprint resolution and aspect ratio of the cross-linked elastomeric network and exhibited substantial advantages in overcoming the imprinting limitations of traditional cross-linked systems. We believed that this strategy could be extended to thermoplastic materials to overcome chain entanglement and friction induced by a high molecular weight (*20*). In addition, this work demonstrated distinct merits in process applicability, including high structural fidelity, pattern diversity, and large scalability (Fig. 2E). By seamlessly assembling multiple templates with different structural features, a centimeter-scale sample with distinct nanostructures at designated domains was obtained (fig. S14), providing alternative technical solutions for high-resolution nanomanufacturing and flexible lithography.

### Dynamic behavior of thermally induced DHGE networks

**Fig. 4.**
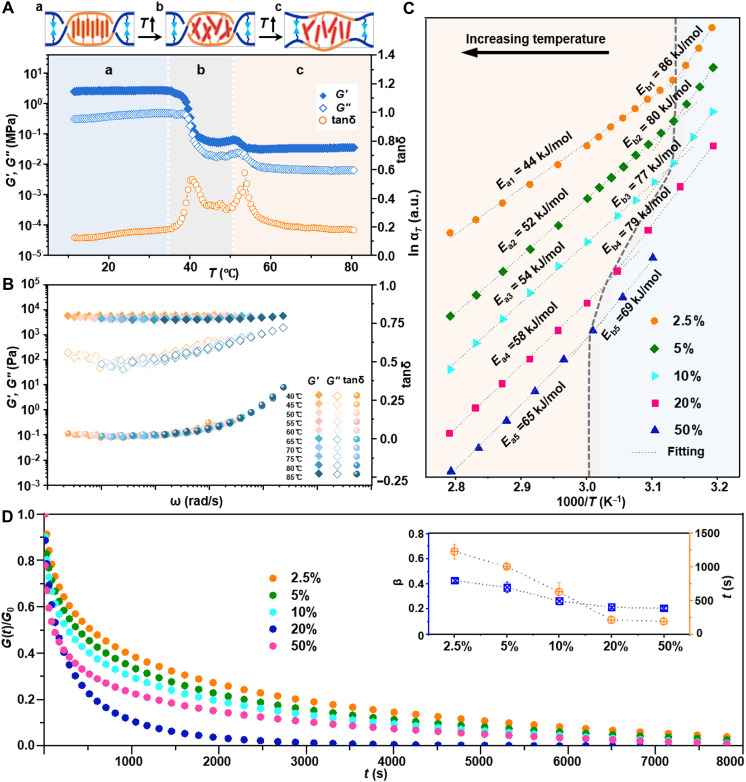
Study on the dynamic behavior of DHGEs. (**A**) Temperature-dependent dynamic mechanics of DHGEs with schematic illustrations of network structure evolution. (**B**) Dynamic modulus spectra of DHGEs with storage moduli *G*′ (solid squares), loss moduli *G*″ (hollow squares), and damping factor tanδ (hollow circles) following TTS with the reference temperature of 60°C. (**C**) Arrhenius plot of the shift factor (α*_T_*) against temperature for samples with varying DAC contents. The slope of the Arrhenius fit represents the activation energy (*E*_a_). a.u., arbitrary units. (**D**) Stress relaxation fitting results of the corresponding DHGE networks. Inset shows the relaxation time and stretching exponent β.

To investigate network reconstruction driven by thermally activated dynamic covalent bonds, we systematically studied the dynamic mechanical behaviors of materials with varying dynamic bond contents. As shown in Fig. 4A, the evolution of mechanical properties with increasing temperature can be divided into three regions: At low temperatures, the gel exhibited stable mechanical properties, with storage modulus *G*′, loss modulus *G*″, and damping factor tanδ remaining relatively constant [Fig. 4A (a)]. Above *T*_m_, the microcrystalline domain melted, softening the gel at 40°C. This process was reflected by a decrease in *G*′ and *G*″, accompanied by an increase in the damping factor, until another modulus plateau was established [Fig. 4A (b)]. As temperature further increased, both moduli decreased whereas the loss factor exhibited a second peak at 55°C, attributed to topological changes caused by dynamic covalent heterogeneous networks [Fig. 4A (c)]. In contrast to the linear polymer above *T*_g_ where chain thermal motion led to flow behavior, the DHGE network maintained solid-like characteristics, providing a broad processing window and preventing uncontrolled deformation and bulk damage during imprint, thus offering distinct advantages for nanoimprint lithography.

The impact of dynamic cross-linking density was measured by the dynamic modulus spectra of DHGEs varying in DAC content. The rheological behaviors of the samples were analyzed across a frequency scan range of 0.01 to 100 rad s^−1^, with maximum strain of 0.5%, and temperatures from 40° to 85°C, increasing in 5°C increments. Time-temperature superposition (TTS) master curves were constructed at a reference temperature of 60°C based on isothermal frequency sweep data (Fig. 4B and fig. S15). During this process, frequency sweep curves were horizontally shifted by temperature-dependent shift factors α*_T_*. The horizontal shift factor was derived from $G^{*}\left(\omega ,T\right)=G^{*}\left(\omega \alpha_{T},T_{0}\right)$, where G*(ωα*_T_*, *T*_0_) indicated the modulus at the reference temperature *T*_0_ and offset frequency ωα*_T_*. As shown in Fig. 4C, the shift factors (α*_T_*) exhibited two distinct linear regions, indicating a transition in network relaxation mechanisms with a characteristic transition temperature (*T*_trans_). Above *T*_trans_, the DHGE network underwent activated topological reconfiguration, and the transition temperature shifted from 45° to 65°C when the DAC content increased from 2.5 to 50%. In addition, the apparent activation energy, calculated using the Arrhenius equation $\alpha_{T}=Ae^{\frac{E_{a}}{\mathrm{RT}}}$ (where *A* is a constant and *R* is the gas constant, 8.314), varied from 45 to 65 kJ/mol and increased with cross-link concentration.

We performed the stress relaxation experiments on a strain-controlled rheometer in the linear viscoelastic regime at 0.5% strain and measured the modulus decay over time. To quantify the relaxation processes, relaxation data were fit to the Kohlrausch–Williams–Watts (KWW) equation

$$G\left(t\right)=\left(G_{0}-G_{P}\right)\text{exp}-{\left(\frac{t}{t_{\text{KWW}}}\right)}^{\beta }e^{-i\omega t}+G_{P}$$

where τ is the characteristic relaxation time, β is a nonexponential parameter measuring the distribution of relaxation time, *G*_0_ is the initial modulus, and *G*_P_ is the plateau modulus. As shown in Fig. 4D, the relaxation time of the dynamic heterogeneous network could be tuned from 1000 to 200 s by varying the DAC content. The stretching exponent β ranges from 0.2 to 0.4, corresponding to the broader relaxation time distribution associated with segmental dynamics relaxation of the DHGE network (usually <0.6). The parameter β decreased with increasing DAC content, reflecting a more heterogeneous network environment induced by increased dynamic cross-linking.

Above studies revealed the temperature-dependent network reconstruction behavior of DHGEs and its relationship with dynamic covalent cross-linking density, providing molecular-level insights into optimizing thermal imprint processes. By tuning the content of DAC, the transition temperature and relaxation behavior of the network could be effectively regulated, offering opportunities for controlled material processing.

### Bioinspired hierarchical elastomers with a mechanically enhanced nanostructured surface via chain translocated crystallization for impact resistance

**Fig. 5.**
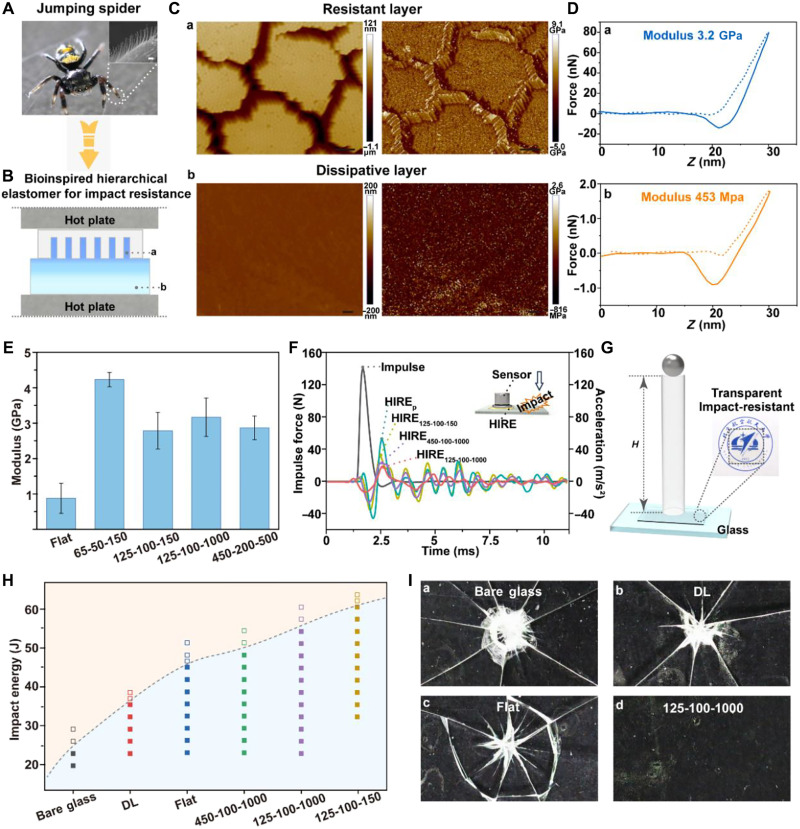
Bioinspired hierarchical elastomers for impact resistance enabled by mechanical enhancement of nanostructures via chain translocated crystallization during imprinting. (**A**) Photograph of a jumping spider and the structural characterization of its feet. The feet surface features the scopulae arranged in tufts with the scopula differentiating into single setae. The inset shows the SEM image of a single seta, which is densely covered by numerous setules. Photo credit: Y.Y., Beihang University. (**B**) Design strategy for HIREs inspired by the jumping spider. The system is constructed through nanoimprinting and interface reconstruction via dynamic covalent bonds to create a hierarchical structure that consists of (a) the impact-resistant layer with enhanced modulus provided by nanostructures and (b) the energy DL. (**C**) AFM characterization showing the morphology and modulus mapping of (a) the impact-resistant layer with nanostructures (125-100-1000) and (b) the energy DL. Scale bars, 500 nm. (**D**) Force-displacement curves for (a) the nanofibers in the impedance layer and (b) the dissipative layer. (**E**) Modulus values of HIRE samples with different nanostructure configurations. (**F**) Damping performance of the HIREs measured by drop weight impact tests. (**G**) Scheme of the falling ball test. The photograph on the right showing the HIRE sample with transparency and excellent impact resistance. (**H**) Energy threshold phase diagram for the glass protection capability of samples (thickness: 1 mm) with various nanostructures. (**I**) Digital images of the glass substrates protected with the DL, Flat, and 125-100-1000 sample after being impacted by the falling ball.

Nature has evolved remarkable solutions for impact protection, particularly evident in arthropods such as the jumping spider. Our investigation of the jumping spider foot structure revealed a sophisticated hierarchical organization where the scopulae differentiated into numerous setae, each densely covered with setules (Fig. 5A and fig. S16). This hierarchical arrangement enabled exceptional impact resistance capabilities critical for the survival of spiders during high-speed jumps and landings. During impact events, the hierarchical setae structures distributed mechanical stresses across multiple length scales while creating a functional gradient that facilitated efficient energy conversion and subsequent dissipation through controlled deformation pathways. Inspired by this biological design principle, we developed HIREs via interface reconstruction using dynamic covalent bonds to weld two functional layers: an impact-resistant layer with high modulus enhanced by nanostructures via nanoimprinting lithography [Fig. 5B (a)] and an energy dissipation layer (DL) [Fig. 5B (b) and fig. S17]. Atomic force microscopy (AFM) characterization revealed distinct differences in morphology and modulus between the two functional layers (Fig. 5C). The resistant layer exhibited well-defined nanostructures with diameters of 100 nm, lengths of 1000 nm, and spacing of 125 nm (designated as 125-100-1000), with a higher modulus values (~3.2 GPa). In contrast, the dissipative layer showed a more uniform, lower-modulus surface (around 453 MPa) designed for energy absorption. The force-displacement curves in Fig. 5D compared the mechanical response of the nanofibers from the resistant layer and the uniform surface of the dissipative layer, demonstrating their complementary mechanical properties that contributed to the hierarchical impact resistance system. Furthermore, a series of impact-resistant elastomers (HIREs) with various nanostructured configurations was fabricated. These nanostructured surfaces demonstrated considerable increases in modulus compared to flat samples that had no nanostructures (Fig. 5E). When the diameter of nanostructures decreased to 50 nm, as in the 65-50-150 sample, the modulus reached ~4.2 GPa, five times higher than that of the flat sample. We attribute this mechanical enhancement to chain translocated crystallization in nanochannels during the imprinting process.

Next, we investigated the energy dissipation performance of HIREs through drop weight impact tests. As shown in Fig. 5F, the HIREs with various nanostructured configurations demonstrated effective shock absorption due to viscoelastic deformation and intermolecular friction within the hierarchical structures. Specifically, the HIRE sample of 125-100-1000 exhibited exceptional energy dissipation capability, reducing an external impact force of 120 N by up to 83%, which can be attributed to its high-aspect-ratio and high-density nanostructures. The synergistic effect of efficient energy dissipation and enhanced impact resistance in the HIRE system enabled it to serve as a promising protective material. To evaluate its protective capability, we conducted falling ball tests where a 33-g steel ball was dropped from various heights onto 1-mm-thick samples firmly attached to 1-mm glass substrates (Fig. 5G and fig. S18). Here, we constructed an energy threshold phase diagram illustrating the glass protection capability of different samples (thickness: 1 mm), including the energy DL, flat HIREs without nanostructures (Flat), and HIREs with various nanostructured configurations (125-100-150, 125-100-1000, and 450-100-1000). As shown in Fig. 5H, unprotected glass was shattered at 23 J of impact energy. The nanostructured surface of HIREs considerably increased the impact energy threshold at which glass fracture occurred. For example, the 125-100-150 sample could protect the underlying 1-mm glass substrate from impacts up to 60 J, despite being only 1 mm thick itself.

To visualize the protective effect, we subjected different systems including bare glass, DL, Flat, and 125-100-1000 to a 50-J impact energy (Fig. 5I). The digital images of fractured glass substrates provided comprehensive insight into the protective performance and energy dissipation mechanisms of different samples. For bare glass [Fig. 5I (a)], fracture was concentrated at the impact point, with the center shattered into numerous fine glass fragments radiating outward in a star pattern. The glass substrate protected by the DL [Fig. 5I (b)] and Flat [Fig. 5I (c)] showed an effective improvement in impact resistance, with fewer fragments at the impact center and larger, more distinct crack patterns. Notably, the flat sample exhibited a distinctive circular crack pattern around the impact point, suggesting more effective distribution of impact energy across a wider area. In contrast, the 125-100-1000 sample [Fig. 5I (d)] with high-modulus nanostructures on the surface prevented glass fracture entirely. Furthermore, the nanostructures also endowed the material with excellent optical transparency, which achieved an integration of high transparency and impact resistance. This thin, elastic protective material showed promising applications in electronic displays and personal safety equipment.

## DISCUSSION

In this work, we developed high-performance elastomer nanoimprinting systems by chain translocated crystallization in nanochannels. Our elastomers exhibited ultrahigh imprinting resolution with sub–10-nm features. The surface structures spanned across seven orders of magnitude in length scale (from sub–10 nm to millimeters) and achieved aspect ratios exceeding 100:1. The dynamic covalent reconstruction process enhanced network mobility and dynamic disentanglement, thereby facilitating efficient chain translocation into nanochannels. Moreover, the crystalline phase further limited the entropy-driven shape recovery and ensured the fidelity of the structure. This approach was successfully extended from gels to various elastomer systems including polyurethanes and acrylates and exhibited excellent versatility. Moreover, inspired by jumping spider hierarchical setae, we constructed HIREs, featuring the nanostructured impact-resistant layer and energy DL. The nanostructures substantially enhanced the surface modulus to 4.2 GPa compared to flat elastomers due to the chain translocated crystallization while simultaneously improving optical transparency, which effectively distributed stress and provided shock absorption through viscoelastic deformation and intermolecular friction within the hierarchical structures. We further simulated bioinspired nanostructures with systematically varied diameters and aspect ratios, successfully replicating high transparency, robust hydrophobicity, and enhanced adhesive properties similar to those observed in biological systems (fig. S19). These results highlight the broad application potential of this material system in the construction of bioinspired nanostructured surfaces.

To further broaden the applicability of this paradigm, future efforts could be directed toward two key considerations: designing materials with lower interfacial adhesion to enable more universal demolding techniques and optimizing the dynamic chemistry to fine-tune the balance between rapid processing and long-term material stability. Addressing these aspects will be an important step toward scaling the technology for diverse industrial settings. We foresee that this advancement in cross-linked elastomeric network imprinting holds promise for bioinspired interface engineering, flexible electronics, and energy technologies.

## MATERIALS AND METHODS

### Materials

DMA, 2,2′-diethoxyacetophenone (DEOP), SMA, poly(ethylene glycol)-*block*-poly(propylene glycol)-*block*-poly(ethylene glycol) [PEG-PPG-PEG; average number-average molecular weight (*M*_n_) ~ 12,600] and *N*,*N*′-methylenebisacrylamide (BIS), hydrogenated 4,4′-methylenediphenyl diisocyanate (HMDI), and dibutyltin dilaurate (DBTDL) were purchased from Aladdin. 2-Hydroxyethyl acrylate (HEA), 2-[2-(2-methoxyethoxy)ethoxy]ethyl acrylate (TEEA), triethylamine, methacryloyl chloride, and EGDMA were purchased from Sigma-Aldrich. NaHCO_3_, NaCl, butyl acrylate (BA), ethyl acrylate (EA), dibenzoyl peroxide (BPO), polytetramethylene ether glycol (PTMEG; *M*_n_ ~ 1000), dichloromethane (CH_2_Cl_2_), *N*,*N*-dimethylacetamide (DMAc), and triethanolamine (TEA) were acquired from Innochem. l-Cystine, sodium hydroxide, methanol, acryloyl chloride, 1,6-hexanediol (HDO), and bis(2-hydroxyethyl) disulfide (HEDS) were purchased from Tokyo Chemical Industry (TCI).

### Synthesis of DAC

Sodium hydroxide (4.12 g, 103 mmol) and l-cystine (5.57 g, 23.16 mmol) were dissolved in methanol (150 ml). Acryloyl chloride (4.53 ml, 55.74 mmol) was added dropwise at 0°C. The solution was stirred at room temperature for 12 hours, and the reaction mixture was filtered. The filtrate was gradually added to cold diethyl ether with vigorous stirring. The obtained precipitant was filtered and vacuum dried for 2 days at 40°C.

### Synthesis of bis(2-(methacryloyloxy)ethyl) disulfide

HEDS (2.0 g, 12.97 mmol) was dispersed in 30 ml of dichloromethane. Then, triethylamine (5.4 ml, 38.85 mmol) was added to the mixture and stirred until a clear solution was obtained. Methacryloyl chloride (3.14 ml, 32.44 mmol) was added dropwise to the solution under ice-water bath conditions. The reaction was allowed to proceed for 24 hours at room temperature under stirring. Afterward, the reaction mixture was filtered to remove the solid. The obtained filtrate underwent a series of washing steps, using a saturated sodium bicarbonate water solution, deionized water, and a saturated sodium chloride water solution. The organic phase was treated with anhydrous magnesium sulfate to absorb water and separated through filtration. Last, the solvent was evaporated under reduced pressure to yield the final product.

### Synthesis of DHGEs

DMA (3.0 g) and DAC (0.02 g) were dispersed in 2.38 g of deionized water and stirred to obtain the hydrophilic precursor. Subsequently, 5.18 g of SMA, 0.08 g of PEG-PPG-PEG, and 50 μl of DEOP were mixed to form the oleophilic precursor. The two precursors were mixed and homogenized at a speed of 18,000 rpm for 10 min to form a precursor. Last, the DHGE was synthesized by in situ phase-separated polymerization under ultraviolet (UV) light (365 nm) for 2 hours and named as DHGE-*x* (where *x* denotes the DAC mass fraction relative to DMA mass). Other types of dynamic heterogeneous hydrogels were also prepared by regulating the types of hydrophilic monomers, including HEA and TEEA.

### Synthesis of DHAEs

To prepare the precursor, 0.5 g of EA, 2.0 g of SMA, 0.02 g of bis(2-(methacryloyloxy)ethyl) disulfide (MASS), and 0.02 g of BPO were mixed and stirred for an hour at 50°C. Then, the obtained solution was transferred to a mold, and a cross-linked solid polymer was obtained at 70°C after 24 hours.

### Synthesis of DHPEs

First, 0.5 g of PTMEG was introduced into a round-bottom flask and dried at 120°C under vacuum for 2 hours to remove the moisture. Subsequently, the system temperature was lowered to 60°C. Under an argon atmosphere, 0.62 g of HEDS and dry DMF were injected into the flask via a syringe. The mixture was then reacted with HMDI under continuous stirring, followed by DBTDL as a catalyst to initiate the polymerization reaction. After 12 hours of chain-extending reaction, 0.22 g of TEA was added into the solution. Following a brief prereaction interval, the resultant solution was transferred to a Teflon mold and the system was then maintained at 45°C to facilitate cross-linking and solvent evaporation for several days until the sample reached a constant weight. In addition, a control sample was obtained by adding HDO to the prepolymer instead of HEDS.

### Fabrication of micro/nanostructure elastomers

The micro/nanostructures were fabricated using various templates spanning multiple size scales. At the nanoscale, anodic aluminum oxide templates were purchased from Topmembranes Technology Co. Ltd., whereas the microscale patterns (circular: 3 μm, square: 5 μm, triangular: 50 μm, and BUAA patterns: 700 μm) were created using etched silicon wafers from Suzhou Yanke Micro-nano Technology Co. Ltd. Linear patterns were fabricated using 3D printing technology. The samples were placed on the template surface and then transferred to a compression molding machine. The system was heated to different temperatures depending on the dynamic transition temperature of the elastomer network while applying a low pressure of 0.2 MPa and maintained for 30 min to ensure complete filling of the structural gaps and adequate stress relaxation through chain segment motion. For the DHGE system, a humid environment was maintained during the heating process to prevent gel dehydration. After the thermal pressing process, the system was cooled to room temperature before removal from the hot press to allow crystallization and structural fixation, followed by demolding to obtain various structured patterns. The anodic aluminum oxide (AAO) templates were removed by wet etching using sodium hydroxide solution. To ensure a mild etching environment without vigorous heat release or chemical reactions that could compromise structural integrity, the NaOH concentration was set at 5 wt %. The etching time depends on the NaOH concentration and template dimensions. For example, AAO templates with an area of 2 cm by 2 cm and aspect ratio below 5:1 require ~3 hours for complete etching, whereas templates with higher aspect ratios require around 10 hours. This mild wet etching ensures high fabrication yield by avoiding structural damage caused by strong interfacial adhesion and stress concentration that is inherent in physical demolding processes.

### Fabrication of large-scale nanostructure elastomers

The large-area (4 cm by 4 cm) sample with multiple nanostructures was fabricated via nanoimprinting using a 2 × 2 array composed of four different anodic aluminum oxide templates. Specifically, the anodic aluminum oxide templates were selected with pore pitch-diameter-depth parameters of 100-50-5000, 125-100-150, 450-200-500, and 450-200-2000, respectively. Using DHPEs as the imprinting material, the entire fabrication procedure followed the protocol detailed in the “Fabrication of micro/nanostructure elastomers” section.

### Fabrication of HIREs

HIREs consisted of an impact-resistant layer and an energy DL. The impact-resistant layer with various structural configurations was fabricated via nanoimprint lithography on the DHPE surface. The energy DL was synthesized using 0.5 g of PTMEG, 0.2314 g of HEDS, 0.5247 g of HMDI, and 3 μl of DBTDL following the DHPE preparation procedure. The two functional layers were then welded together through dynamic covalent reconstruction at 110°C under pressure for 1 hour, with the overall thickness controlled by silicone spacers.

### Differential scanning calorimetry

DSC measurements were performed using a calorimeter (DSC 3, Mettler Toledo, Switzerland) at a heating speed of 5°C min^−1^, from 5° to 60°C under nitrogen flow, for characterization of the phase-separation behavior of SMA. The glass transition temperature (*T*_g_) was determined by the temperature corresponding to the maximum slope nearby the transition.

### Compressive tests

Compressive tests were performed on a tensile-compressive tester (Tensilon HZ-1004D, Hengzhun Instrument Technology Co., China). Cylinder-shaped samples (10-mm diameter by 5-mm height) were measured with an applied compression strain of 0 to 60% at a constant rate of 5 mm min^−1^. The elastic modulus (*E*) was calculated from the slope of the stress-strain curves between 20 and 30% deformation. All measurements were performed in triplicate (*n* = 3).

### Atomic force microscopy

The height images and modulus images were obtained by tapping mode scanning of an atomic force microscope (Bruker) using a rectangular silicon cantilever beam with a resonance frequency of 300 kHz.

### SEM characterization

A scanning electron microscope (JSM-7500F, JEOL, Japan) and an environmental scanning electron microscope (Quanta 250 FEG, FEI, Czech Republic) were used for micro/nanostructure characterization.

### Nuclear magnetic resonance

^1^H nuclear magnetic resonance (NMR) and ^13^C NMR spectra were acquired on a Bruker AVANCE 400 (400 MHz) spectrometer at room temperature. For DAC samples, measurements were performed in HDO with chemical shifts referenced to the residual solvent peak at 4.79 parts per million (ppm), whereas MASS samples were measured in CDCl_3_ with the residual proton signal at 7.26 ppm. Sample concentrations were prepared at 1.0 and 10.0 mM for ^1^H and ^13^C NMR measurements, respectively.

### Rheological characterization

The rheological measurements were implemented using an Anton Paar MCR-302 rheometer equipped with a temperature control system. Dynamic frequency sweep tests were conducted at angular frequencies (ω) ranging from 0.1 to 100 rad s^−1^ with a constant strain amplitude (γ) of 0.1% at designated temperatures. Temperature sweep measurements were carried out from 10° to 80°C at a heating rate of 2°C min^−1^ for DHGEs while maintaining a constant frequency of 10 rad s^−1^ and a strain amplitude of 0.1%. The frequency master curves were plotted by TTS with reference temperatures of 100° and 65°C for DHPEs and DHGEs, respectively. The stress relaxation measurements of DHGEs with varying DAC contents were carried out at 65°C with a strain amplitude of 0.5%. For all DHGE samples, measurements were conducted in water to prevent dehydration.

### Shock absorption tests

The impulse signal (the impulse force) was produced by an impact hammer (PCB Piezotronics Inc.). The response signals were collected by an acceleration sensor.

## Supplementary Materials

### This PDF file includes:

Supplementary Text Figs. S1 to S19 Table S1 References

## References

[1] Y. Peng, C. M. Serfass, A. Kawazoe, Y. Shao, K. Gutierrez, C. N. Hill, V. J. Santos, Y. Visell, L. C. Hsiao, Elastohydrodynamic friction of robotic and human fingers on soft micropatterned substrates. Nat. Mater. 20, 1707–1711 (2021). 33927390 10.1038/s41563-021-00990-9

[2] S. Chen, L. Sun, X. Zhou, Y. Guo, J. Song, S. Qian, Z. Liu, Q. Guan, E. M. Jeffries, W. Liu, Y. Wang, C. He, Z. You, Mechanically and biologically skin-like elastomers for bio-integrated electronics. Nat. Commun. 11, 1107 (2020). 32107380 10.1038/s41467-020-14446-2 PMC7046662

[3] L. Zhang, H. Chen, Y. Guo, Y. Wang, Y. Jiang, D. Zhang, L. Ma, J. Luo, L. Jiang, Micro–nano hierarchical structure enhanced strong wet friction surface inspired by tree frogs. Adv. Sci. 7, 2001125 (2020). 10.1002/advs.202001125 PMC7578903 33101853

[4] A. Chortos, J. Liu, Z. Bao, Pursuing prosthetic electronic skin. Nat. Mater. 15, 937–950 (2016). 27376685 10.1038/nmat4671

[5] Y. Ru, M. Liu, Superwetting gels: Wetting principles, applications, and challenges. ACS Nano 19, 7583–7600 (2025). 39970347 10.1021/acsnano.4c17507

[6] S. Feng, P. Zhu, H. Zheng, H. Zhan, C. Chen, J. Li, L. Wang, X. Yao, Y. Liu, Z. Wang, Three-dimensional capillary ratchet-induced liquid directional steering. Science 373, 1344–1348 (2021). 34529472 10.1126/science.abg7552

[7] M. Liu, S. Wang, L. Jiang, Nature-inspired superwettability systems. Nat. Rev. Mater. 2, 17036 (2017).

[8] D. Hwang, C. Lee, X. Yang, J. M. Pérez-González, J. Finnegan, B. Lee, E. J. Markvicka, R. Long, M. D. Bartlett, Metamaterial adhesives for programmable adhesion through reverse crack propagation. Nat. Mater. 22, 1030–1038 (2023). 37349397 10.1038/s41563-023-01577-2

[9] J. Chai, Y. Ru, Y. Jia, Y. Yang, H. Zhang, L. Chen, T. Zhao, M. Liu, Friction memory ionogels with hysteretic sticky-slippery transition via thermolocking the metastable state. Adv. Mater. 37, e2416250 (2025). 40018829 10.1002/adma.202416250

[10] J. Kim, S. Kim, T. Yun, J. H. Kim, C. Son, Y. Lee, K. Kim, H. E. Lee, N. Kim, S. Kim, Shape memory polymer surfaces with controllable roughness for multiscale switchable dry adhesion. Nat. Commun. 16, 4954 (2025). 40436828 10.1038/s41467-025-60220-7 PMC12119915

[11] M. Wang, C. Li, S. Napolitano, D. Wang, G. Liu, Quantifying and modeling the crystallinity of polymers confined in nanopores. ACS Macro Lett. 13, 908–914 (2024). 38990566 10.1021/acsmacrolett.4c00287

[12] V. Narasimhan, R. H. Siddique, J. O. Lee, S. Kumar, B. Ndjamen, J. Du, N. Hong, D. Sretavan, H. Choo, Multifunctional biophotonic nanostructures inspired by the longtail glasswing butterfly for medical devices. Nat. Nanotechnol. 13, 512–519 (2018). 29713074 10.1038/s41565-018-0111-5 PMC5992053

[13] S. Y. Chou, P. R. Krauss, P. J. Renstrom, Imprint lithography with 25-nanometer resolution. Science 272, 85–87 (1996).

[14] D. Huang, J. Wu, C. Chen, X. Fu, A. H. Brozena, Y. Zhang, P. Gu, C. Li, C. Yuan, H. Ge, M. Lu, M. Zhu, L. Hu, Y. Chen, Precision imprinted nanostructural wood. Adv. Mater. 31, e1903270 (2019). 31592564 10.1002/adma.201903270

[15] S. Liang, C. Yuan, C. Nie, Y. Liu, D. Zhang, W. C. Xu, C. Liu, G. Xu, S. Wu, Photocontrolled reversible solid-fluid transitions of azopolymer nanocomposites for intelligent nanomaterials. Adv. Mater. 36, e2408159 (2024). 39082060 10.1002/adma.202408159

[16] L. J. Guo, Nanoimprint lithography: Methods and material requirements. Adv. Mater. 19, 495–513 (2007).

[17] C. Guo, L. Feng, J. Zhai, G. Wang, Y. Song, L. Jiang, D. Zhu, Large-area fabrication of a nanostructure-induced hydrophobic surface from a hydrophillic polymer. ChemPhysChem 5, 750–753 (2004). 15179734 10.1002/cphc.200400013

[18] B. Wang, M. Sanviti, A. Alegría, S. Napolitano, Molecular mobility of polymers at the melting transition. ACS Macro Lett. 12, 389–394 (2023). 36867860 10.1021/acsmacrolett.3c00003

[19] H. D. Rowland, W. P. King, J. B. Pethica, G. L. Cross, Molecular confinement accelerates deformation of entangled polymers during squeeze flow. Science 322, 720–724 (2008). 18832609 10.1126/science.1157945

[20] H. Schift, Nanoimprint lithography: An old story in modern times? A review. J. Vac. Sci. Technol. B 26, 458–480 (2008).

[21] Z. Hao, A. Ghanekarade, N. Zhu, K. Randazzo, D. Kawaguchi, K. Tanaka, X. Wang, D. S. Simmons, R. D. Priestley, B. Zuo, Mobility gradients yield rubbery surfaces on top of polymer glasses. Nature 596, 372–376 (2021). 34408328 10.1038/s41586-021-03733-7

[22] P. Choi, P. F. Fu, L. J. Guo, Siloxane copolymers for nanoimprint lithography. Adv. Funct. Mater. 17, 65–70 (2007).

[23] J. L. Shamshina, P. Berton, Ionic liquids as designed, multi-functional plasticizers for biodegradable polymeric materials: A mini-review. Int. J. Mol. Sci. 25, 1720 (2024). 38338998 10.3390/ijms25031720 PMC10855424

[24] E. Thoms, Z. Song, K. Wang, S. Napolitano, Simple model to predict the adsorption rate of polymer melts. Phys. Rev. Lett. 132, 248101 (2024). 38949357 10.1103/PhysRevLett.132.248101

[25] H. Tian, C. Bi, Z. Li, C. Wang, B. Zuo, Metastable polymer adsorption dictates the dynamical gradients at interfaces. Macromolecules 56, 4346–4353 (2023).

[26] B. Yang, F. Cai, S. Huang, H. Yu, Athermal and soft multi-nanopatterning of azopolymers: Phototunable mechanical properties. Angew. Chem. Int. Ed Engl. 132, 4064–4071 (2020). 10.1002/anie.201914201 31823474

[27] H.-C. Scheer, N. Bogdanski, M. Wissen, S. Möllenbeck, Impact of glass temperature for thermal nanoimprint. J. Vac. Sci. Technol. B 25, 2392–2395 (2007).

[28] C. Probst, C. Meichner, K. Kreger, L. Kador, C. Neuber, H. W. Schmidt, Athermal azobenzene-based nanoimprint lithography. Adv. Mater. 28, 2624–2628 (2016). 26822954 10.1002/adma.201505552

[29] G. Nian, Z. Chen, X. Bao, M. W. M. Tan, Y. Kutsovsky, Z. Suo, Natural rubber with high resistance to crack growth. Nat. Sustain. 8, 692–701 (2025).

[30] W. Kuhn, F. Grün, Beziehungen zwischen elastischen Konstanten und Dehnungsdoppelbrechung hochelastischer Stoffe. Kolloid-Zeitschrift 101, 248–271 (1942).

[31] Y. Mao, B. Talamini, L. Anand, Rupture of polymers by chain scission. Extreme Mech. Lett. 13, 17–24 (2017).

[32] L. Chen, C. Zhao, J. Huang, J. Zhou, M. Liu, Enormous-stiffness-changing polymer networks by glass transition mediated microphase separation. Nat. Commun. 13, 6821 (2022). 36357428 10.1038/s41467-022-34677-9 PMC9649666

[33] X. Zhao, L.-M. Peng, Y. Chen, X.-J. Zha, W.-D. Li, L. Bai, K. Ke, R.-Y. Bao, M.-B. Yang, W. Yang, Phase change mediated mechanically transformative dynamic gel for intelligent control of versatile devices. Mater. Horiz. 8, 1230–1241 (2021). 34821916 10.1039/d0mh02069a

[34] H. Meng, P. Xiao, J. Gu, X. Wen, J. Xu, C. Zhao, J. Zhang, T. Chen, Self-healable macro-/microscopic shape memory hydrogels based on supramolecular interactions. Chem. Commun. 50, 12277–12280 (2014). 10.1039/c4cc04760e 25126654

[35] J. Chung, J. W. Chung, R. D. Priestley, S.-Y. Kwak, Confinement-induced change in chain topology of ultrathin polymer fibers. Macromolecules 51, 4229–4237 (2018).

[36] C. Wu, L. Li, Unified description of transportation of polymer chains with different topologies through a small cylindrical pore. Polymer 54, 1463–1465 (2013).

[37] Y. Jeong, S. Shin, H. Choi, S. Kim, J. Kim, S. Kwon, K.-Y. Kim, S.-H. Lee, Y.-G. Jung, Y. T. Cho, Fabrication of nano-micro hybrid structures by replication and surface treatment of nanowires. Crystals 7, 215 (2017).

[38] G. Chen, S. A. Soper, R. L. McCarley, Free-standing, erect ultrahigh-aspect-ratio polymer nanopillar and nanotube ensembles. Langmuir 23, 11777–11781 (2007). 17929951 10.1021/la701502m

[39] M. S. Kim, S. Shin, W. Y. Kim, S. H. Lee, S. R. Park, S. Kim, Y. T. Cho, Formation of multiscale porous surfaces via evaporation-induced aggregation of imprinted nanowires with highly viscous photocurable materials. Int. J. Precis. Eng. Manuf. 26, 217–225 (2025).

[40] W. Zhang, H. Wang, A. T. L. Tan, A. Sargur Ranganath, B. Zhang, H. Wang, J. Y. E. Chan, Q. Ruan, H. Liu, S. T. Ha, D. Wang, V. K. Ravikumar, H. Y. Low, J. K. W. Yang, Stiff shape memory polymers for high-resolution reconfigurable nanophotonics. Nano Lett. 22, 8917–8924 (2022). 36354246 10.1021/acs.nanolett.2c03007

[41] S. H. Kang, B. Pokroy, L. Mahadevan, J. Aizenberg, Control of shape and size of nanopillar assembly by adhesion-mediated elastocapillary Interaction. ACS Nano 4, 6323–6331 (2010). 21038896 10.1021/nn102260t

[42] H. Wang, Q. Ruan, H. Wang, S. D. Rezaei, K. T. P. Lim, H. Liu, W. Zhang, J. Trisno, J. Y. E. Chan, J. K. W. Yang, Full color and grayscale painting with 3D printed low-index nanopillars. Nano Lett. 21, 4721–4729 (2021). 34019769 10.1021/acs.nanolett.1c00979

[43] B. Pokroy, S. H. Kang, L. Mahadevan, J. Aizenberg, Self-organization of a mesoscale bristle into ordered, hierarchical helical assemblies. Science 323, 237–240 (2009). 19131625 10.1126/science.1165607

[44] J. Y. E. Chan, Q. Ruan, M. Jiang, H. Wang, H. Wang, W. Zhang, C.-W. Qiu, J. K. W. Yang, High-resolution light field prints by nanoscale 3D printing. Nat. Commun. 12, 3728 (2021). 34140502 10.1038/s41467-021-23964-6 PMC8211842

[45] P. Zhu, Q. Song, S. Bhagwat, F. Mayoussi, A. Goralczyk, N. Nekoonam, M. Sanjaya, P. Hou, S. Tisato, F. Kotz-Helmer, D. Helmer, B. E. Rapp, Generation of precision microstructures based on reconfigurable photoresponsive hydrogels for high-resolution polymer replication and microoptics. Nat. Commun. 15, 5673 (2024). 38971797 10.1038/s41467-024-50008-6 PMC11227548

[46] L. Xue, X. Xiong, B. P. Krishnan, F. Puza, S. Wang, Y. Zheng, J. Cui, Light-regulated growth from dynamic swollen substrates for making rough surfaces. Nat. Commun. 11, 963 (2020). 32075979 10.1038/s41467-020-14807-x PMC7031321

[47] D. Chen, C. Ni, L. Xie, Y. Li, S. Deng, Q. Zhao, T. Xie, Homeostatic growth of dynamic covalent polymer network toward ultrafast direct soft lithography. Sci. Adv. 7, eabi7360 (2021). 34669482 10.1126/sciadv.abi7360 PMC8528418

[48] Y. Zhu, J. Li, T. Ma, X. Gao, K. Li, X. Ma, X. Jiang, Self-wrinkling-induced mechanically adaptive patterned surface of photocuring coating for abrasion resistance. Adv. Mater. 37, e2414352 (2024). 39718221 10.1002/adma.202414352

[49] H. F. Chan, R. Zhao, G. A. Parada, H. Meng, K. W. Leong, L. G. Griffith, X. Zhao, Folding artificial mucosa with cell-laden hydrogels guided by mechanics models. Proc. Natl. Acad. Sci. U.S.A. 115, 7503–7508 (2018). 29967135 10.1073/pnas.1802361115 PMC6055139

[50] M. J. P. Biggs, M. Fernandez, D. Thomas, R. Cooper, M. Palma, J. Liao, T. Fazio, C. Dahlberg, H. Wheadon, A. Pallipurath, A. Pandit, J. Kysar, S. J. Wind, The functional response of mesenchymal stem cells to electron-beam patterned elastomeric surfaces presenting micrometer to nanoscale heterogeneous rigidity. Adv. Mater. 29, 1702119 (2017). 10.1002/adma.201702119 PMC7391933 28861921

[51] J. De-La-Cuesta, E. González, A. J. Moreno, A. Arbe, J. Colmenero, J. A. Pomposo, Size of elastic single-chain nanoparticles in solution and on surfaces. Macromolecules 50, 6323–6331 (2017).

[52] M. Rubinstein, R. H. Colby, in *Polymer Physics* (Oxford Univ. Press, 2003).

[53] J. Hwang, D. G. Lee, H. Yeo, J. Rao, Z. Zhu, J. Shin, K. Jeong, S. Kim, H. W. Jung, A. Khan, Proton transfer hydrogels: Versatility and applications. J. Am. Chem. Soc. 140, 6700–6709 (2018). 29767509 10.1021/jacs.8b03514

[54] J. Chen, S. Yao, B. Wang, Q. Yu, B. Xue, P. Yin, Polymer films’ residual stress attenuation from the supramolecular complexation with ultra-small nanoparticles for high resolution nanoimprint lithography. Angew. Chem. Int. Ed Engl. 64, e202416759 (2025). 39714372 10.1002/anie.202416759

[55] S. Zeng, Y. Liu, S. Li, K. Shen, Z. Hou, A. P. Chooi, A. T. Smith, Z. Chen, L. Sun, Smart laser-writable micropatterns with multiscale photo/moisture reconstructible structure. Adv. Funct. Mater. 31, 2009481 (2021).

[56] J. Zou, S. Wu, J. Chen, X. Lei, Q. Li, H. Yu, S. Tang, D. Ye, Highly efficient and environmentally friendly fabrication of robust, programmable, and biocompatible anisotropic, all-cellulose, wrinkle-patterned hydrogels for cell alignment. Adv. Mater. 31, e1904762 (2019). 31566289 10.1002/adma.201904762

[57] J. Kim, J. Yoon, R. C. Hayward, Dynamic display of biomolecular patterns through an elastic creasing instability of stimuli-responsive hydrogels. Nat. Mater. 9, 159–164 (2010). 20023633 10.1038/nmat2606

[58] Z. Zhao, C. Li, Z. Dong, Y. Yang, L. Zhang, S. Zhuo, X. Zhou, Y. Xu, L. Jiang, M. Liu, Adaptive superamphiphilic organohydrogels with reconfigurable surface topography for programming unidirectional liquid transport. Adv. Funct. Mater. 29, 1807858 (2019).

[59] Z. Zhao, S. Zhuo, R. Fang, L. Zhang, X. Zhou, Y. Xu, J. Zhang, Z. Dong, L. Jiang, M. Liu, Dual-programmable shape-morphing and self-healing organohydrogels through orthogonal supramolecular heteronetworks. Adv. Mater. 30, e1804435 (2018). 30328637 10.1002/adma.201804435

[60] K. Gong, L. Hou, P. Wu, Hydrogen-bonding affords sustainable plastics with ultrahigh robustness and water-assisted arbitrarily shape engineering. Adv. Mater. 34, e2201065 (2022). 35261086 10.1002/adma.202201065

[61] P. Ditsche-Kuru, E. S. Schneider, J.-E. Melskotte, M. Brede, A. Leder, W. Barthlott, Superhydrophobic surfaces of the water bug Notonecta glauca: A model for friction reduction and air retention. Beilstein J. Nanotechnol. 2, 137–144 (2011). 21977425 10.3762/bjnano.2.17 PMC3148060

[62] A. F. Pomerantz, R. H. Siddique, E. I. Cash, Y. Kishi, C. Pinna, K. Hammar, D. Gomez, M. Elias, N. H. Patel, Developmental, cellular and biochemical basis of transparency in clearwing butterflies. J. Exp. Biol. 224, jeb237917 (2021). 34047337 10.1242/jeb.237917 PMC8340268

[63] K. Xu, P. Zi, X. Ding, Learning from biological attachment devices: Applications of bioinspired reversible adhesive methods in robotics. Front. Mech. Eng. 17, 43 (2022).

