# Supplementary material for: Sub–10-nm imprint lithography on elastomers by chain translocated crystallization in nanochannels
Source: Sci Adv. 2026 May 22;12(21):eaec3829. doi: 10.1126/sciadv.aec3829 (PMC13196756; doi:10.1126/sciadv.aec3829)
Supplement: Supplementary file 1 — Supplementary Text Figs. S1 to S19 Table S1 References [file sciadv.aec3829_sm.pdf]

Science Advances
AAAS

Supplementary Materials for

Sub–10-nm imprint lithography on elastomers by chain translocated crystallization in nanochannels

Yingchao Yang *et al.*

Corresponding author: Mingjie Liu, liumj@buaa.edu.cn

Sci. Adv. **12**, eaec3829 (2026)
DOI: 10.1126/sciadv.aec3829

This PDF file includes:

Supplementary Text
Figs. S1 to S19
Table S1
References

Supplementary Text

1. The evaluation of D_{min} for passage of chains with degree of polymerization N

The translocation behaviors of polymer chains through nanochannels are reasonably analyzed using scaling theory based on the Flory-type of free energy. Considering the real experimental situations, we suppose that the nanochannels are cylindrical pores with a diameter D and their length is much larger than the size of the polymer chains. The Flory free energy of the single chains F has two contributions: an excluded volume contribution F_{ex} arising from the monomer-monomer and monomer-solvent interactions, and an elastic term F_{el} from chain stretching. The excluded volume interaction is related to the total number of contacts within the polymer chains under confinement and can be written as

$$F_{\text{ex}} = \frac{vN^2b^3}{D^2R}$$

where v is the excluded volume parameter, N the degree of polymerization, b the Kuhn monomer length, and R represents the linear size of single chain (note that the energy is measured in units of $k_BT \equiv 1$, and k_B the Boltzmann constant, T the temperature, and we also set $v \equiv 1$ in the following discussion). The elastic energy strongly depends on the chain topological structure. For example, for linear and randomly branched chains, one has

$$F_{\text{el}} = \frac{R^2}{R_0^2}$$

where R_0 is the polymer size at the ideal situation (for example, the ideal mean radius of gyration). It is well known that for linear chains, $R_0^2 \simeq b^2N$, and for randomly branched chains, $R_0^2 \simeq b^2N^{1/2}$. Minimizing the free energy $F=F_{el}+F_{ex}$ with respect to R gives the size of polymer chains of polymerization N under confinement D, i.e., for linear chains, $R \simeq bD^{-2/3}N$, and for randomly branched chains, $R \simeq bD^{-2/3}N^{5/6}$ (*51*). For single crosslinked chains (i.e., elastic single chains), the situation becomes a bit different, as one has to take into account the elastic contribution from crosslinking. In this case, the Flory energy reads

$$F = \kappa R^2 + \frac{N^2b^3}{D^2R}$$

where κ is a constant depending on the fraction of crosslinks (*52*). Minimizing the free energy of elastic single chains gives $R \simeq b\kappa^{-1/3}D^{-2/3}N^{2/3}$.

Now we calculate the minimum diameter $D_{\min}$ that allows passage of chains with degree of polymerization N of different topological structures. The highest possible packing condition inside the pore imposes a restriction on the chain volume fractions, i.e.,

$$\phi = \frac{Nb^3}{D_{\min}^2 R} \simeq 1$$

which gives the critical condition to evaluate $D_{\min}$. Inserting the expression of R with respect to D just obtained gives $D_{\min}$ for chains of different topological structures, i.e., for linear chains, $D_{\min} \simeq b$ irrelevant to the degree of polymerization N, for randomly branched chains $D_{\min} \simeq bN^{1/8}$, and for crosslinked single chains $D_{\min} \simeq bN^{1/4}$. In contrast, the degree of polymerization of an infinite polymer network can be treated as infinite, therefore it is reasonable to expect the corresponding $D_{\min}$ as infinity.

2. Network design strategies for overcoming entropy-driven elastic recovery

The dynamic network also contributes to stress dissipation through bond exchange reactions. In practice, due to the time-dependent nature of stress relaxation through bond exchange, complete stress dissipation cannot be achieved within the limited processing window, leading to gradual creep at room temperature owing to entropy-driven network recovery and compromising the structural fidelity. To further restrain the entropy-driven shape recovery, we incorporated strong interactions such as hydrogen bonding and crystallization to form heterogeneous phases within the network. These heterogeneous domains provide higher energy barriers and restrict chain segment mobility. The restricted chain mobility results in stress being stored within the network, thereby thermodynamically stabilizing the imprinted structures (fig. S1).

3. Current methods for fabricating micro/nanostructures on elastomer surfaces

Elastomers exhibit remarkable stability and soft-elastic properties, serving as vital functional materials across diverse industries. Surface structure fabrication on these

elastomers at micro- and nanoscale not only preserves their bulk intrinsic mechanical and physical properties but also enables customized interfacial functionalities on demand, facilitating applications in tunable mechanical responses, optical diffraction, and wettability control. As is well known, casting represents a conventional approach for constructing nanostructures. However, sub-100 nm features with high aspect ratios (>10:1) remain challenging, where the complexity of precursor wetting behavior and residual stress accumulation during solidification become increasingly critical at diminishing scales (*53, 54*). Moreover, the liquid-to-solid process becomes invalid for achieving surface patterning on pre-formed structures under realistic conditions. While electron beam direct writing offers ultrahigh resolution and precise pattern control, its practical implementation in elastomer fabrication remains challenging due to the poor radiation tolerance of most polymers, particularly in solvent-containing systems. The radiation sensitivity of elastomers leads to a conflict between resolution and aspect ratios, where achieving high resolution down to tens of nanometers unavoidably compromises aspect ratios with shallow features (*50*). These trade-off requirements of two attributes, combined with low throughput and high processing costs, limit its widespread application. Recent progress in materials science has witnessed the emergence of stimulus-induced growth as a promising approach for fabricating surface structures on elastomers (*45-49, 55-57*). In this process, external stimuli such as stress and light alter the local physicochemical properties of materials to induce structural heterogeneity or gradient fields, triggering mass transport and structural evolution that ultimately leads to the formation of specific surface morphologies. However, it suffers from poor morphological controllability due to the coupled influence of multiple parameters during the growth process, typically yielding microscale features with aspect ratios below 2:1. Compared to the aforementioned methods, nanoimprint technology provides a simple and cost-effective approach for large-scale surface structure fabrication with considerable processing flexibility (*32-34, 58-60*). Unlike thermoplastic polymers that can be molded into nanostructures with high resolution and aspect-ratio, the fabrication of fine nanoscale structures on crosslinked elastomers faces significant challenges primarily due to their unique network characteristics. The

kinetically arrested reptation diffusion and entropic elasticity of crosslinked networks not only restrict the accessibility and filling degree of the nanochannels during imprinting but also lead to spontaneous shape recovery upon force removal. These network-induced limitations lead to a significant challenge for imprinting ultrahigh resolution and aspect-ratio nanostructures on elastomers, which are crucial for advanced applications in photonics and surface engineering.

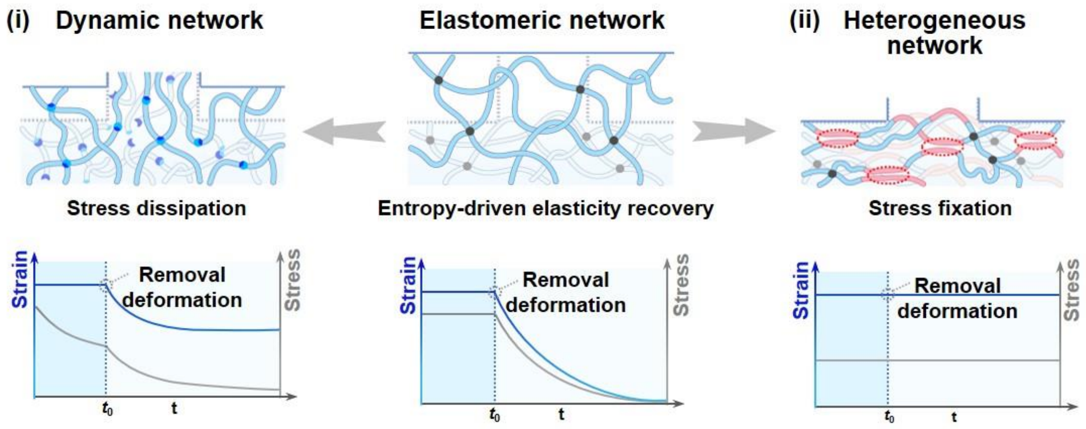

Fig. S1. Network design strategies for overcoming entropy-driven elastic recovery. **(i)** The dynamic network featuring a stress dissipation mechanism, and **(ii)** The heterogeneous network enabling stress fixation. Corresponding stress-strain evolution curves over time are shown in the lower panel.

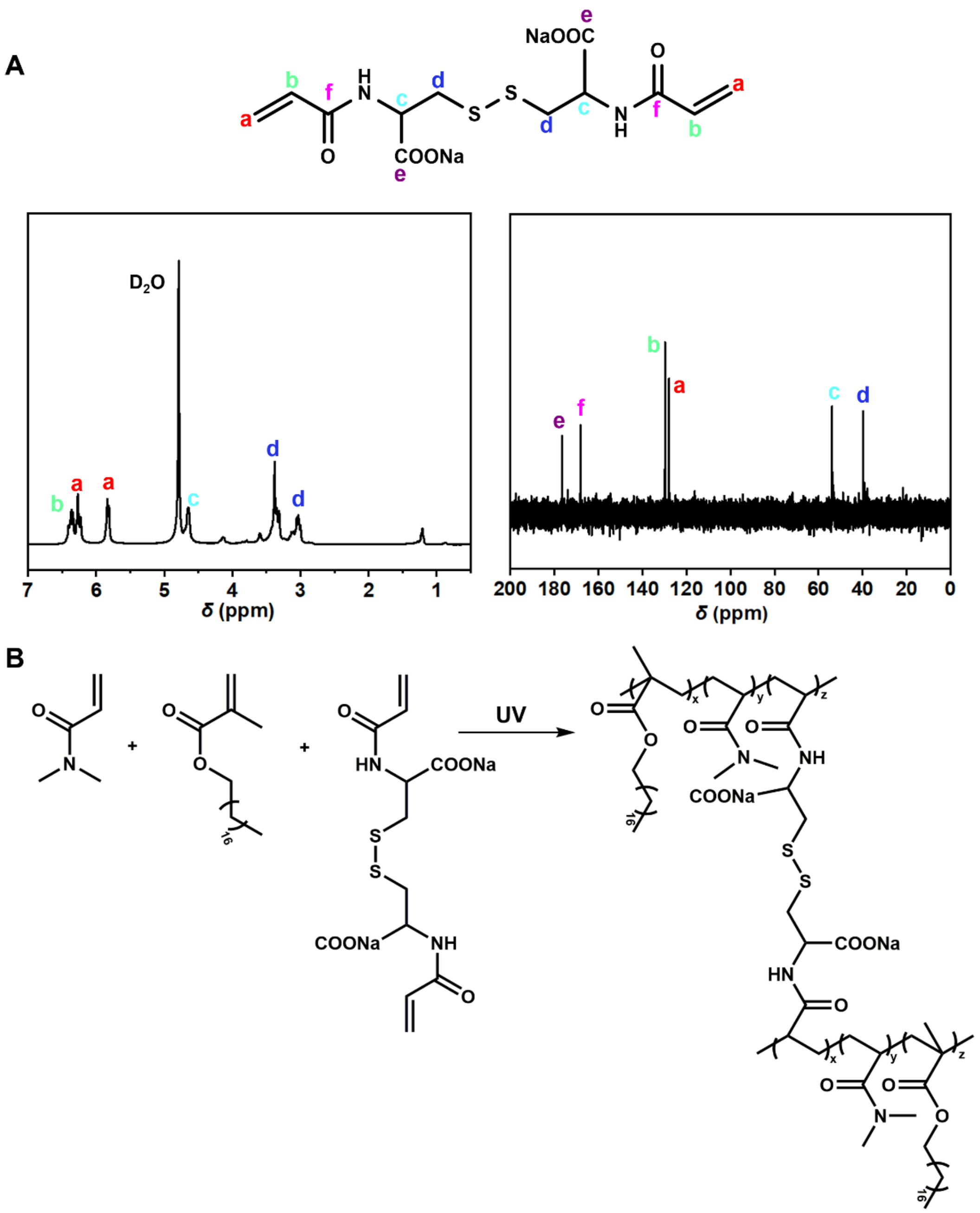

Fig. S2. (A) The chemical structure of DAC and corresponding ^{1}H NMR (left) and ^{13}C NMR (right) spectral characterization. **(B)** Schematic illustration of the in-situ free radical copolymerization process.

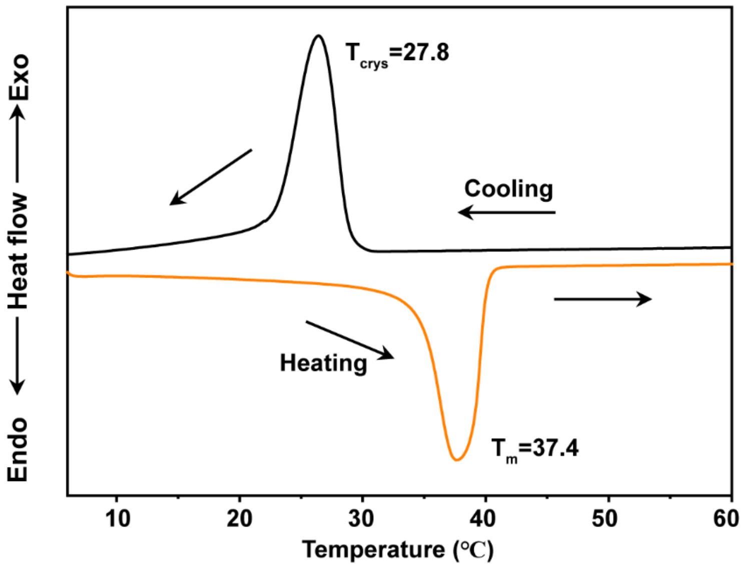

Fig. S3. Differential scanning calorimetry (DSC) curve of the dynamic heterogeneous gels (DHGE) sample.

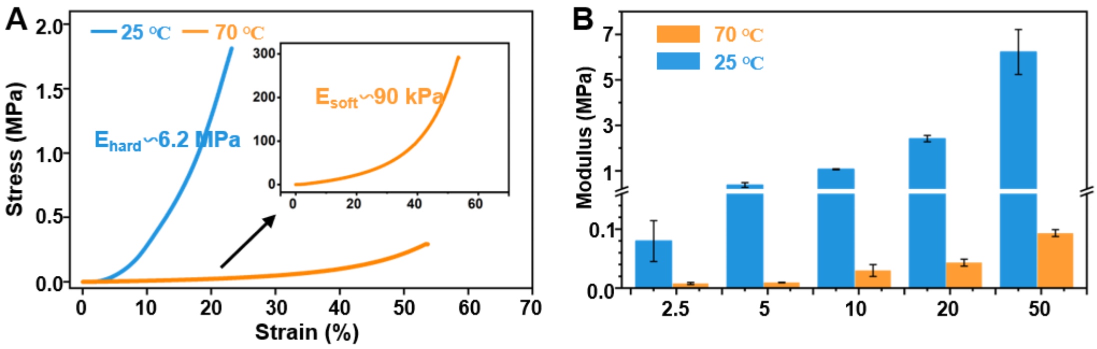

Fig. S4. Mechanical properties of the DHGE at different states and DAC contents. (A) Compressive stress-strain curves at 25°C and 70°C with elastic moduli (E_{hard}=6.2 MPa at 25°C and E_{soft}=90 kPa at 70°C). The inset shows the curve details at low strain. **(B)** The compressive moduli of DHGE-x with varying DAC contents (x = 2.5-50 wt%) at both 25°C and 70°C, demonstrating that increased crosslinking density enhances moduli in both states while maintaining their significant difference. Error bars represent standard deviation (n=3).

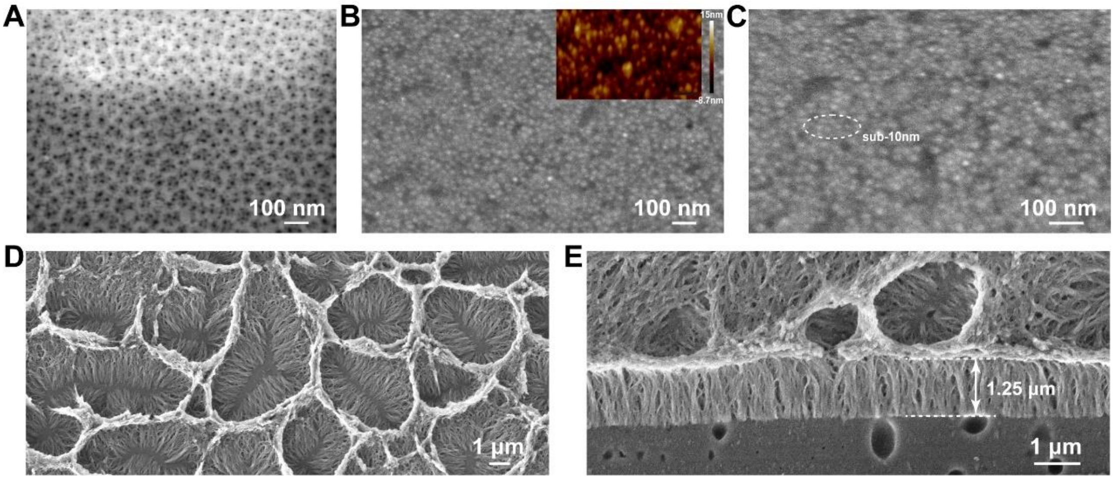

Fig. S5. (A) SEM image of the AAO template. **(B, C)** High-resolution SEM images including sub-10 nm features (The inset in Fig. S5(B) shows the corresponding AFM characterization, scale bar: 100 nm). **(D, E)** SEM images of sub-10 nm features with 100:1 aspect ratio structures: **(D)** top-view and **(E)** side-view.

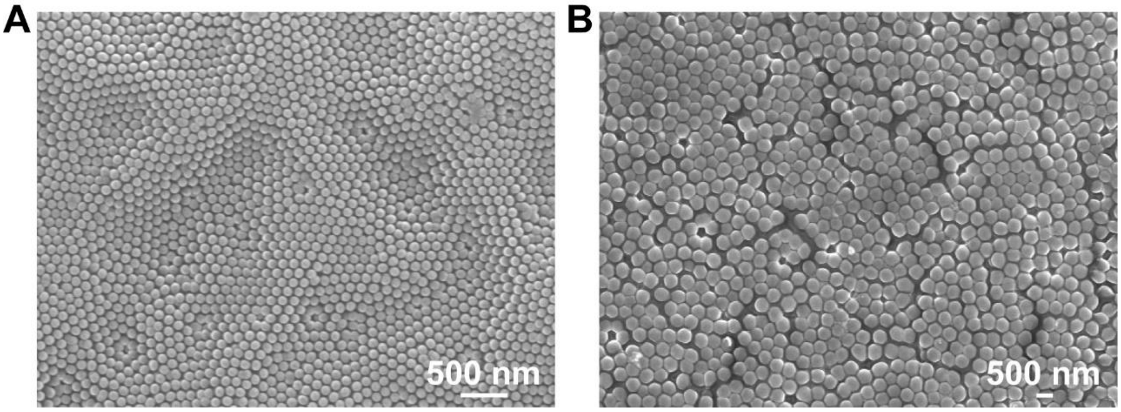

Fig. S6. SEM characterization of imprinted nanostructures with various feature sizes. (A) 100 nm. **(B)** 400 nm.

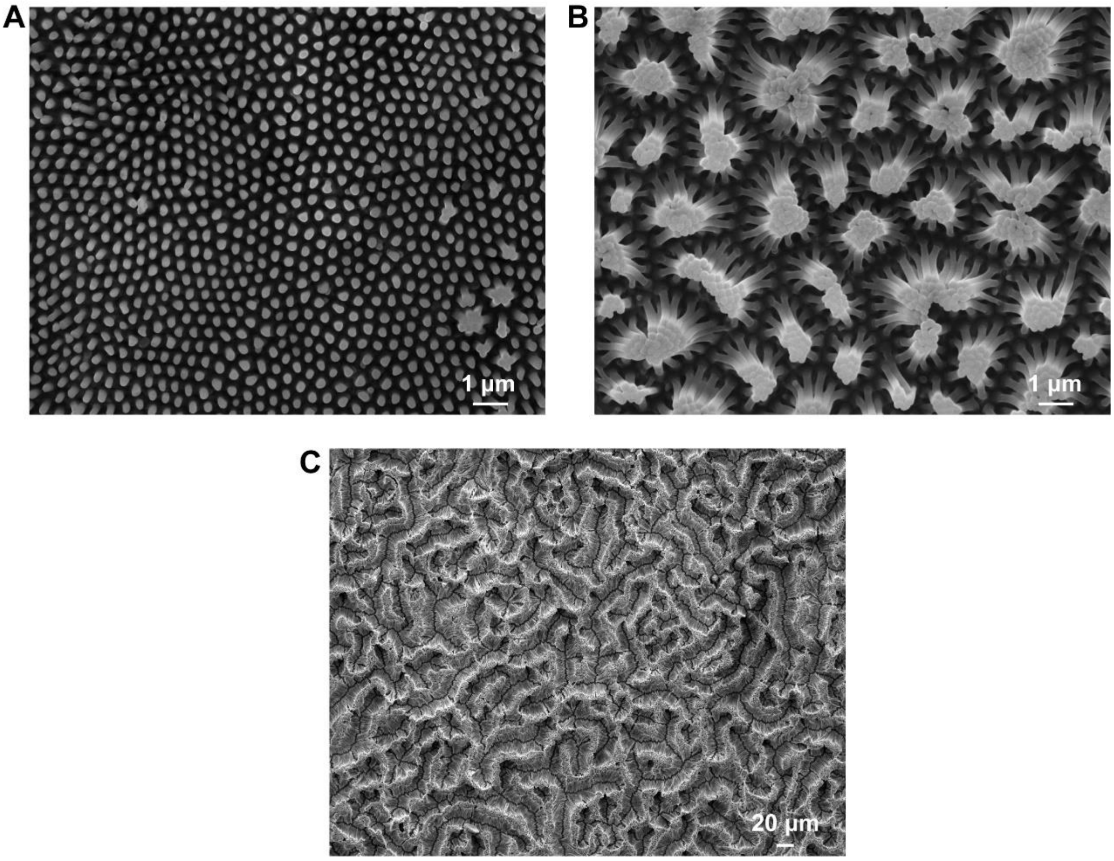

Fig. S7. Low-magnification top-view SEM images with a feature size of 200 nm at different aspect ratios. (A) 5:1. **(B)** 10:1. **(C)** 100:1.

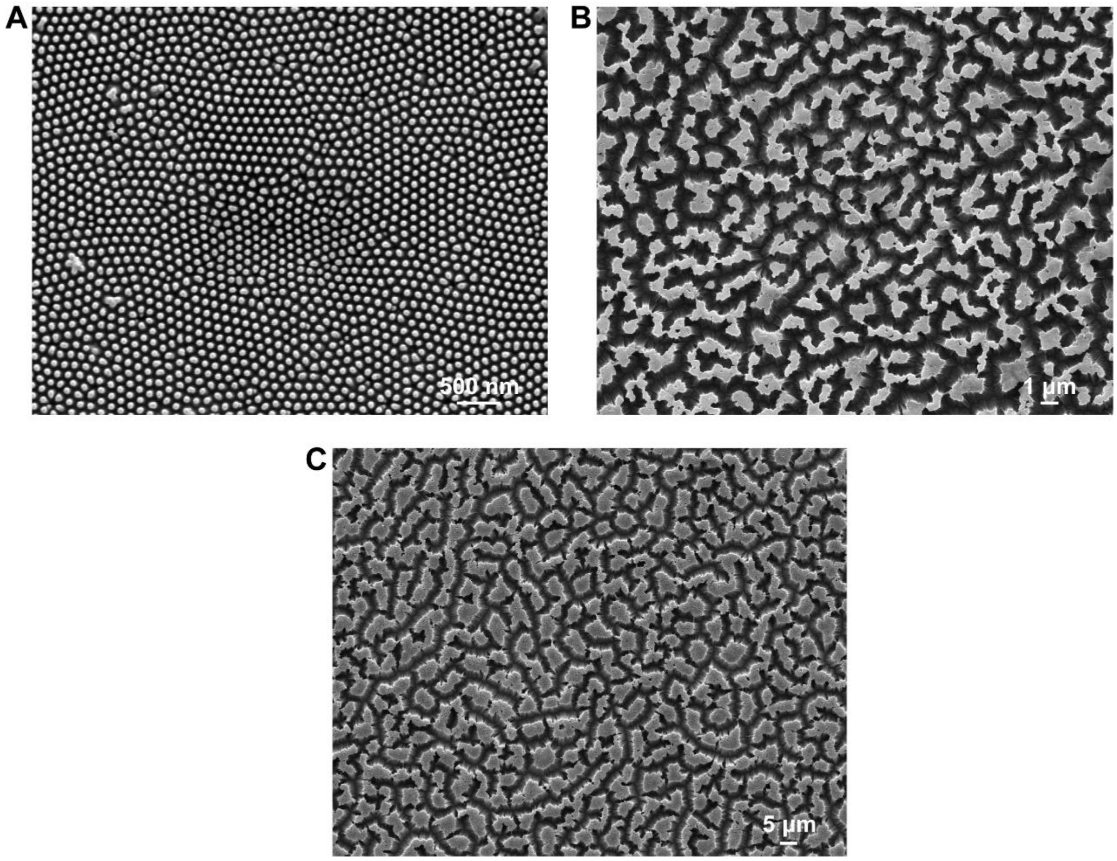

Fig. S8. Low-magnification top-view SEM images with a feature size of 50 nm at different aspect ratios. (A) 3:1. **(B)** 20:1. **(C)** 100:1.

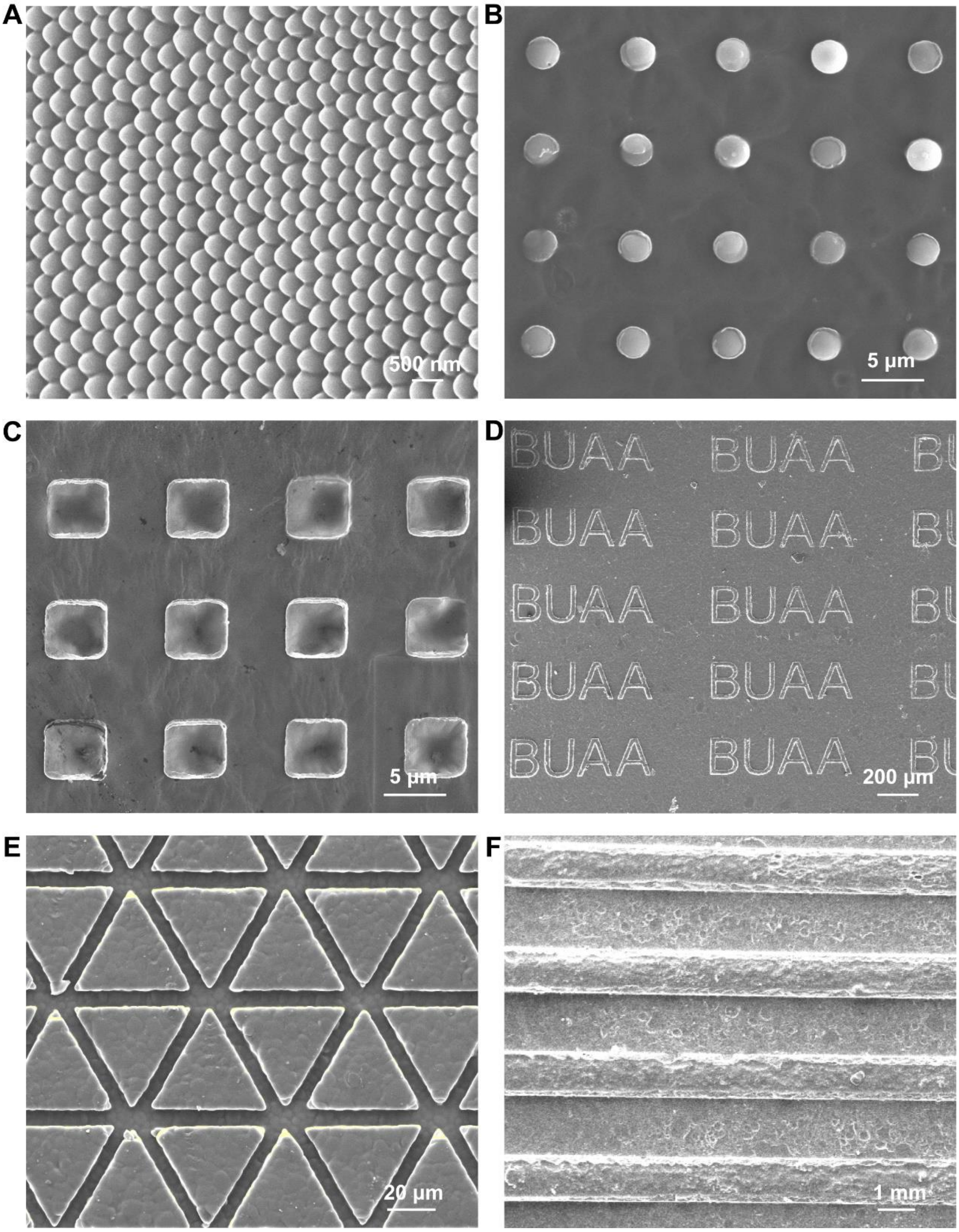

Fig. S9. SEM images showing diversified patterns with various dimensions. (A) V-shaped structures (top diameter: 100 nm, bottom diameter: 450 nm, depth: 400 nm). **(B)** circular pillars (3 μm). **(C)** square pillars (5 μm). **(D)** triangular patterns (50 μm). **(E)** letters "BUAA" (700 μm). **(F)** periodic grooves with millimeter dimensions.

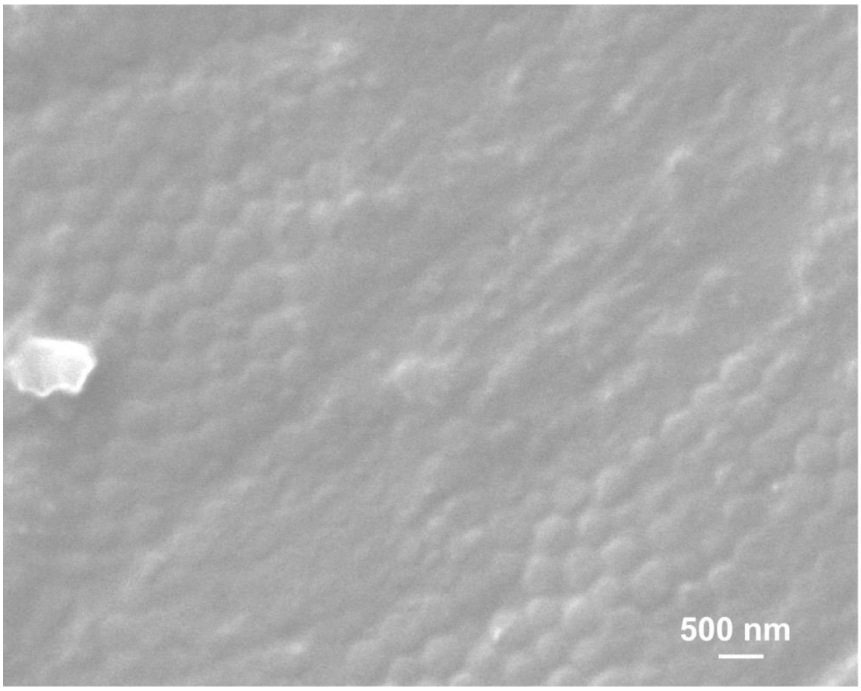

Fig. S10. SEM image showing the imprinting results using EGDMA-based networks with 90-1000 nm patterns, revealing the limited imprinting quality.

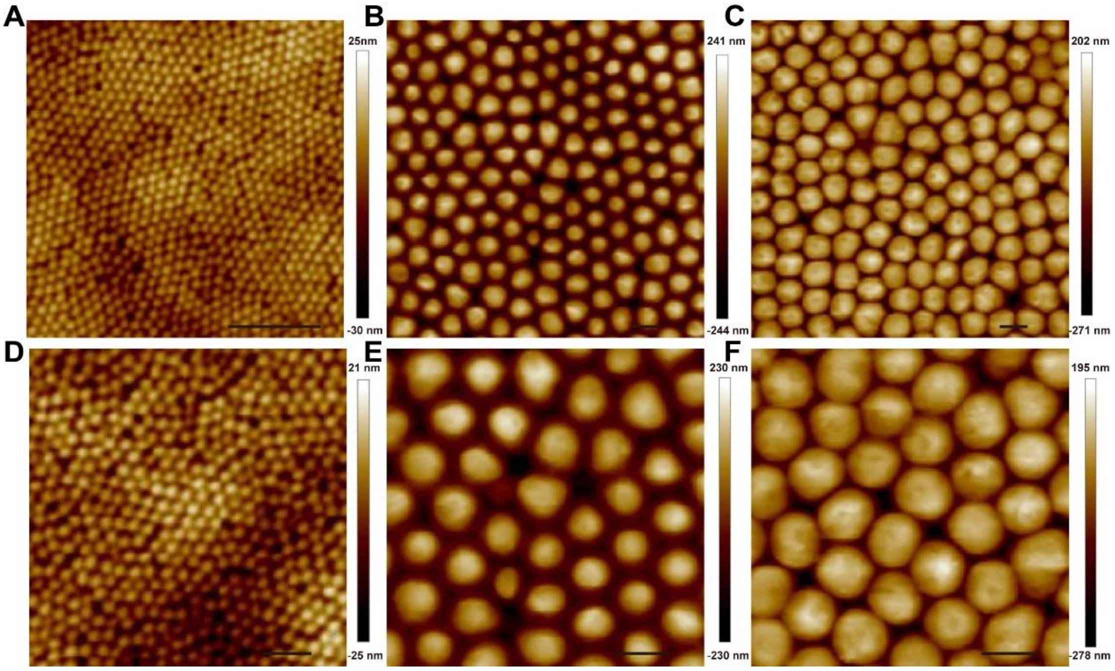

Fig. S11. AFM characterization of patterns fabricated on DHAE with different feature sizes. (A, D) 50 nm, **(B, E)** 200 nm, and **(C, F)** 400 nm. Top row: low-magnification; bottom row: high-magnification. Scale bars: 500 nm except for (D) where it is 200 nm.

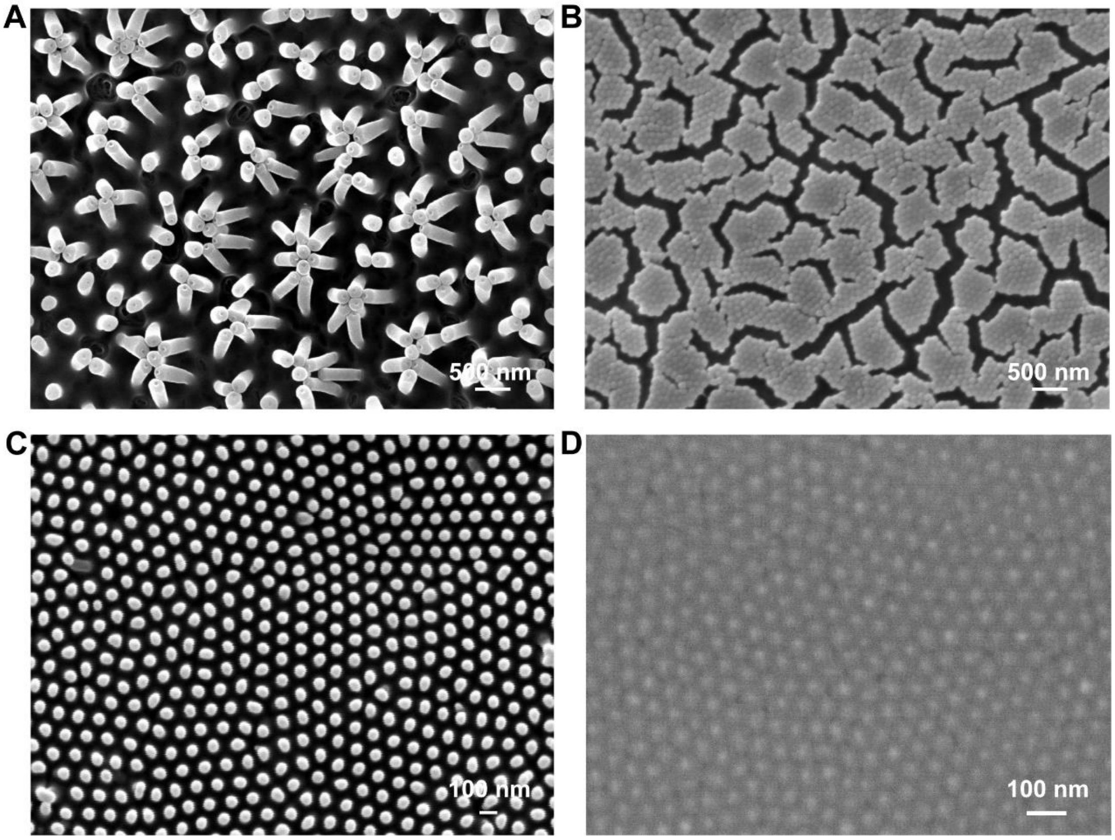

Fig. S12. SEM characterization of patterns fabricated on DHPE with different feature sizes. (A) 200 nm. **(B)** 100 nm. **(C)** 50 nm. **(D)** 20 nm.

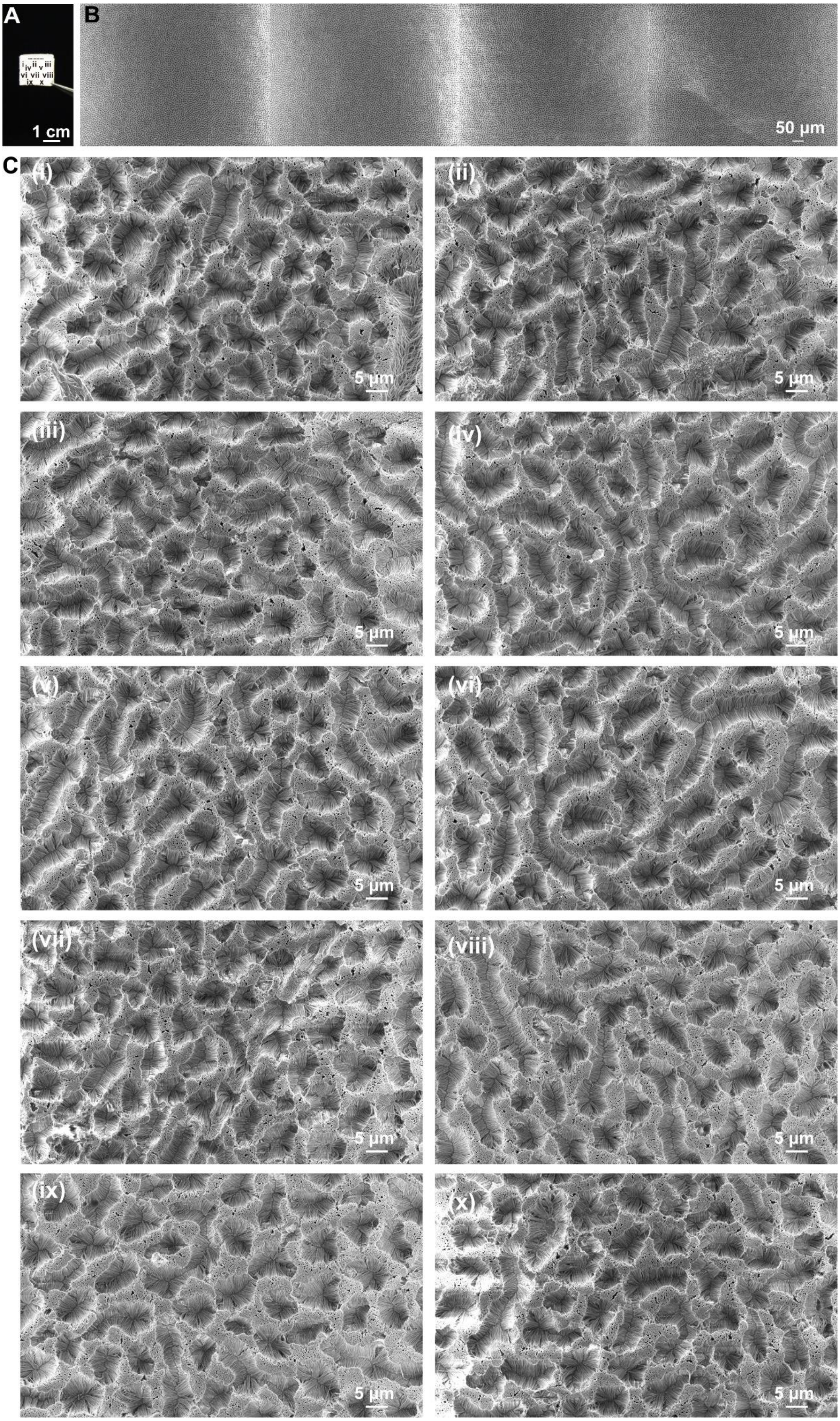
A
i iv ii v iii
vi vii viii
ix x
1 cm
B
50 µm
C
(i)
5 µm
(ii)
5 µm
(iii)
5 µm
(iv)
5 µm
(v)
5 µm
(vi)
5 µm
(vii)
5 µm
(viii)
5 µm
(ix)
5 µm
(x)
5 µm

Fig. S13. Morphological characterization demonstrating fabrication yield across different length scales. (A) Optical image of the DHPE sample with a feature size of 50 nm and aspect ratios of 100:1. Dashed line indicating the scanning path and numbered positions (i-x) marking the ten selected locations distributed across different regions to ensure comprehensive coverage of the sample for microscopic observation. **(B)** Low-magnification SEM image along the scanning path showing uniform structural distribution at the macroscopic scale. **(C)** Representative high-magnification SEM images taken at ten different randomly selected positions (i-x) across the sample, demonstrating consistent structural quality at the microscopic scale.

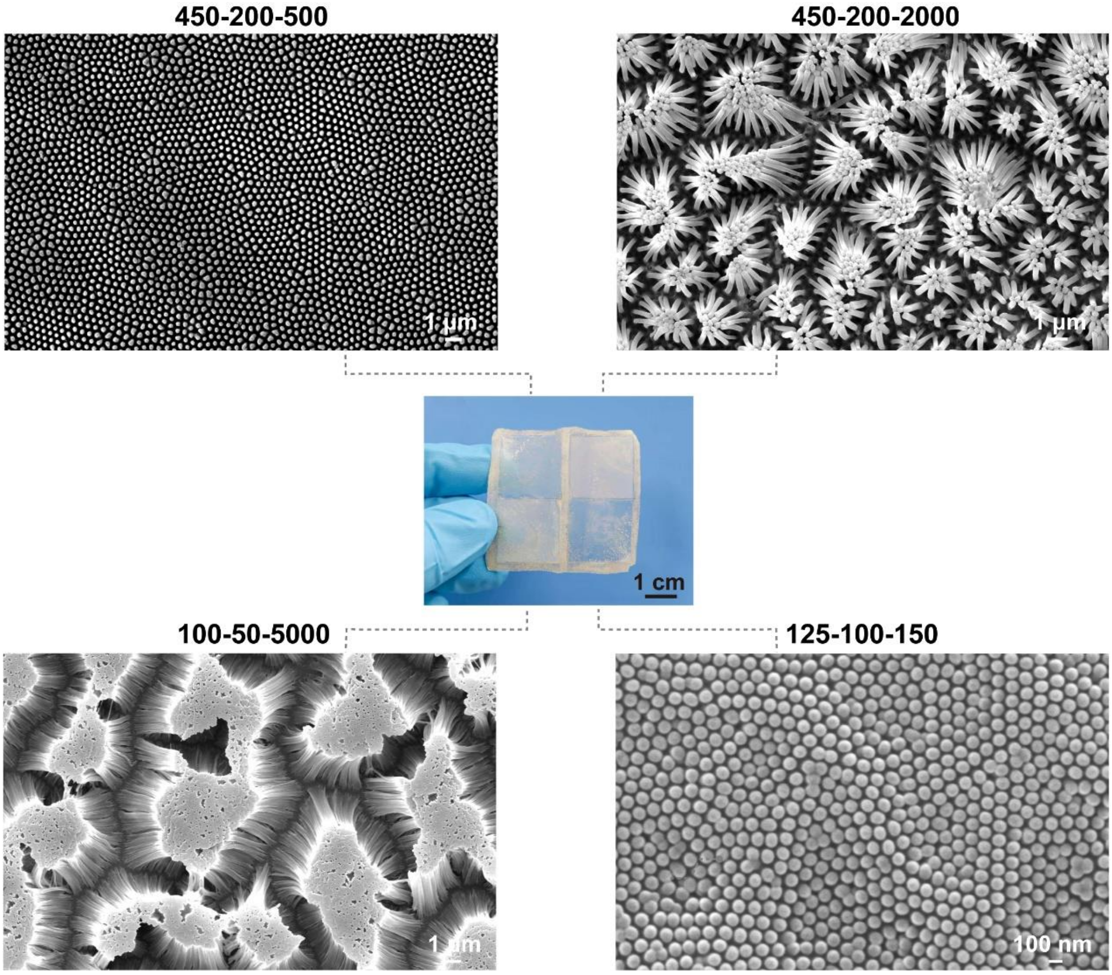

Fig. S14. The 4 cm × 4 cm-scale imprinted sample constructed by AAO templates array with different nanostructures in different regions.

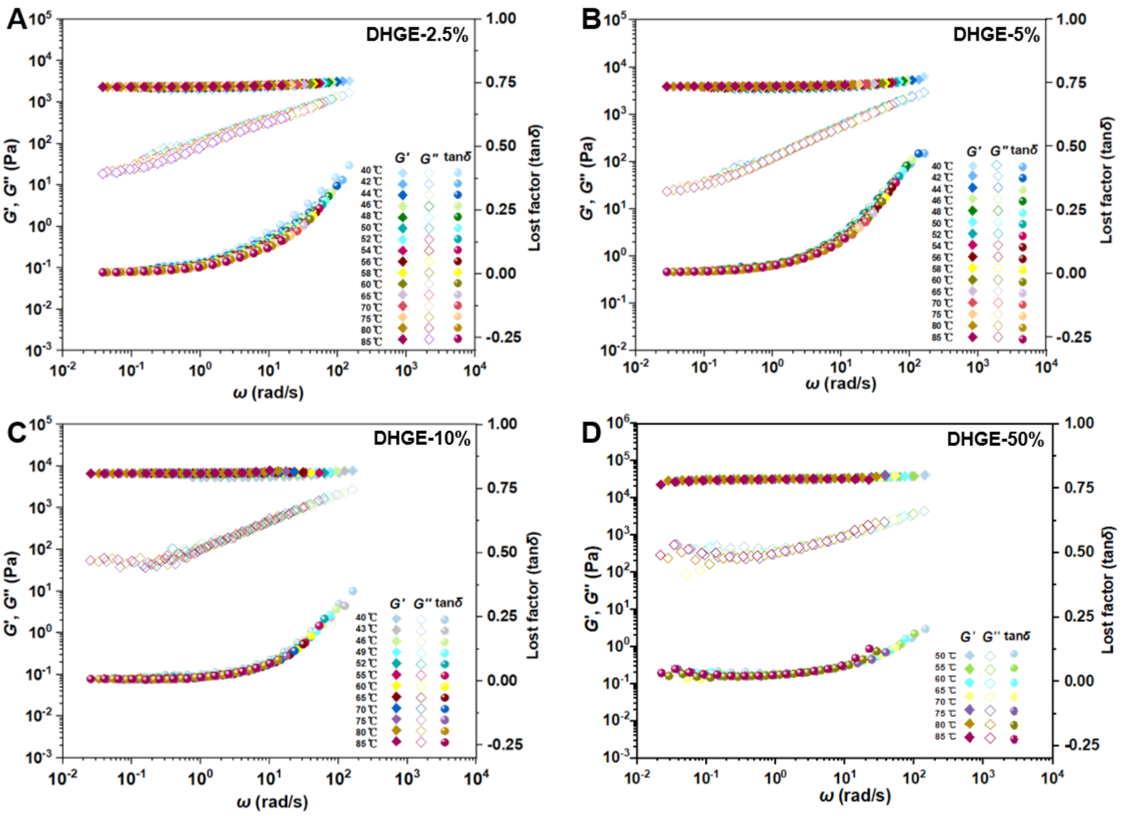

Fig. S15. Rheological master curves showing the frequency dependence of storage modulus (G′) and loss modulus (G″) for DHGE with different mass fractions of DAC relative to DMA monomer. (A) 2.5%. **(B)** 5%. **(C)** 10%. **(D)** 50%. The master curves are constructed by horizontal shifting of isothermal frequency sweep data to a reference temperature of 60°C.

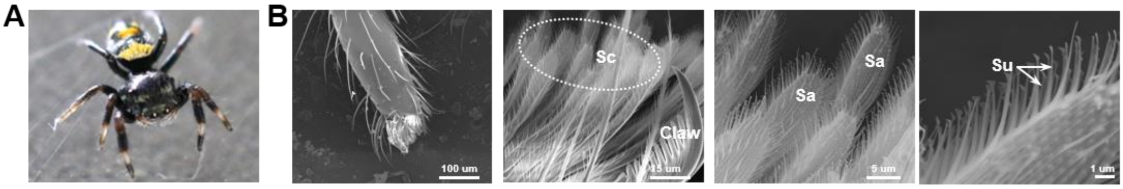

Fig. S16. Photograph of a jumping spider and the structural characterization of its feet. **(A)** Digital photograph of jumping spider. **(B)** Scanning electron micrographs showing progressively magnified views of the tarsal attachment apparatus in the jumping spider. The tarsus of the spider consists of a pair of claws and scopula (Sc). Ventral view of the scopula differentiation into single setae (Sa). The single seta is densely covered by numerous setules (Su). Photo Credit: Yingchao Yang, Beihang University.

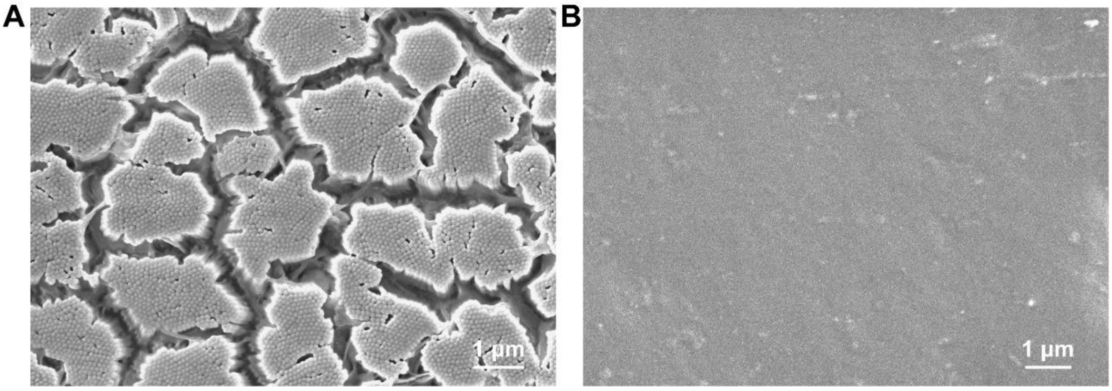

Fig. S17. SEM characterization of the hierarchical impact-resistant elastomers. (A) The impact-resistant layer with nanostructures (125-100-1000). **(B)** The energy dissipation layer.

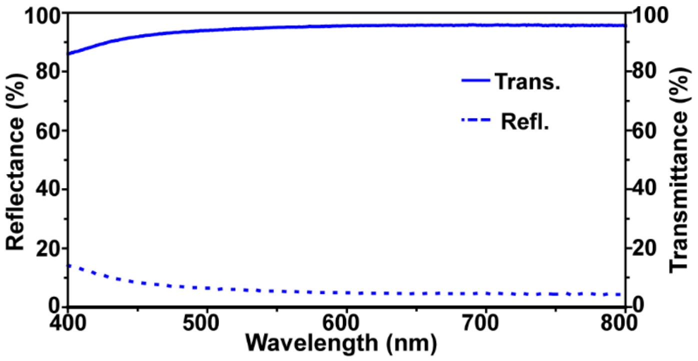

Fig. S18. UV-visible spectroscopy results showing transmittance (Trans.) and reflectance (Refl.) versus wavelength for $DHPE_{125\text{-}50\text{-}150}$.

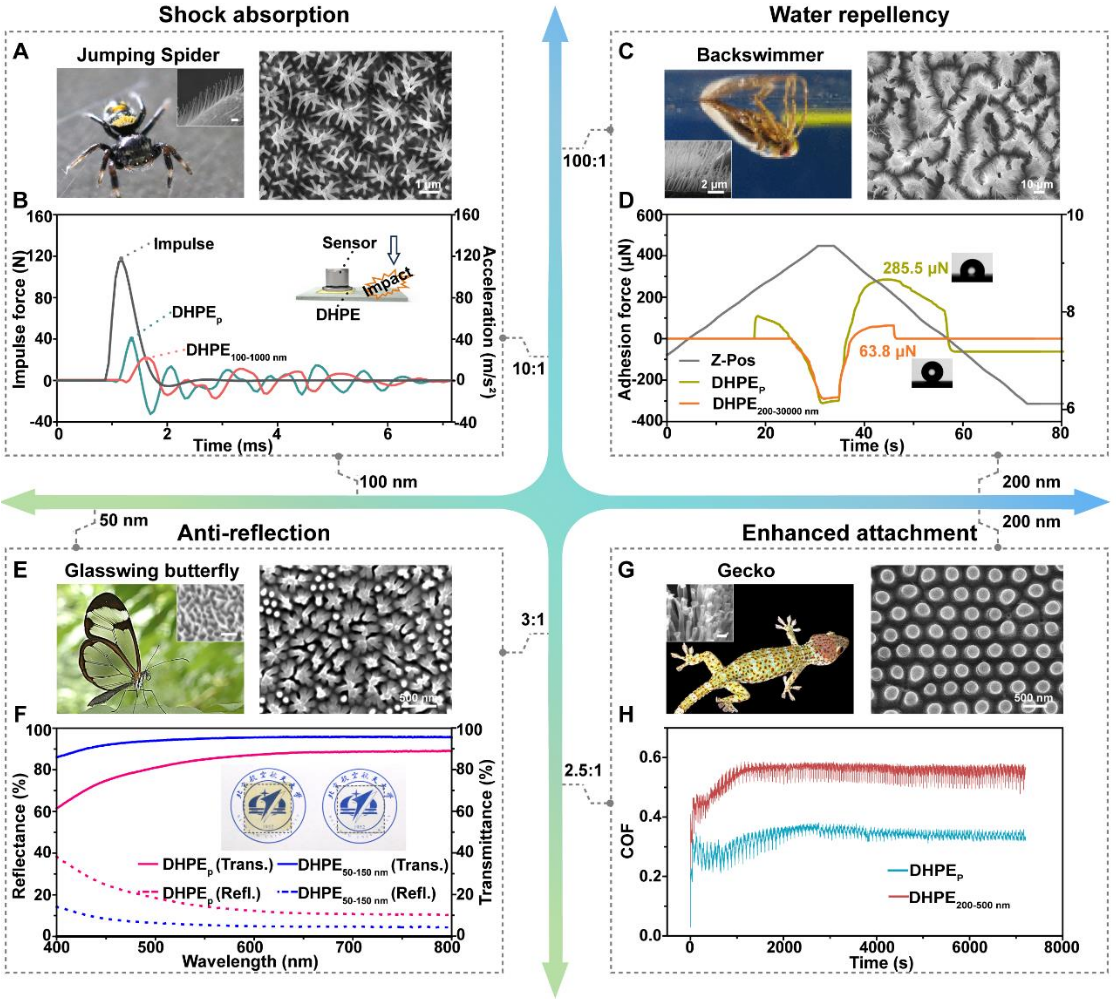

Fig. S19. Fabrication and functional integration of biomimetic nanostructures on crosslinked elastomer surfaces. (A) Spider-inspired shock absorption. Photograph of a jumping spider and SEM images of its leg setae structure (inset), with corresponding SEM morphology of biomimetic $DHPE_{100-1000}$ setae array on the right. Photo Credit: Yingchao Yang, Beihang University. **(B)** Schematic diagram of drop-weight impact test apparatus and impact force-time curves for different samples. **(C)** Backswimmer-inspired water repellency. Photograph of a backswimmer and SEM images of its dorsal setae (inset), with corresponding SEM morphology of biomimetic $DHPE_{200-20000}$ setae array on the right. Photo and morphology of dorsal setae: Adapted from (*61*), under CC BY 2.0 license, published by Beilstein-Institut Forderung der Chemischen Wissenschaft. **(D)** Adhesion force-time curves of $DHPE_{200-20000}$ and $DHPE_P$ during loading and unloading processes; inset shows the contact angle measurements of $DHPE_{200-20000}$ and $DHPE_P$. **(E)** Glasswing butterfly-inspired anti-reflection. Photograph of a glasswing butterfly and SEM images of its wing membrane surface structure (inset), with corresponding SEM morphology of biomimetic $DHPE_{50-150}$ nanopillar array on the right. Photo from pxhere.com (https://pxhere.com/zh/photo/1409533), under CC0 license. Morphology of wing membrane surface structure: Adapted from (*62*), under CC BY 4.0 license (https://creativecommons.org/licenses/by/4.0/), published by The Company of Biologists Ltd. **(F)** UV-visible spectroscopy results showing transmittance (Trans.) and reflectance (Refl.) versus wavelength for $DHPE_{50-150}$ and $DHPE_P$ films; inset shows comparative transparency

photographs. Scale bar: 1 cm. **(G)** Gecko-inspired friction enhancement. Photograph of a gecko and SEM images of its foot pad surface (inset), with corresponding SEM morphology of biomimetic $DHPE_{200\text{-}500}$ nanopillar array on the right. Photo from pixabay.com (https://pixabay.com/photos/gecko-lizard-tokhe-reptile-239812), under CC0 license. Morphology of foot pad surface: Adapted from (*63*), under CC BY 4.0 license (https://creativecommons.org/licenses/by/4.0/), published by Springer Nature. **(H)** Friction coefficient-time curves of $DHPE_{200\text{-}500}$ and $DHPE_{P}$, demonstrating friction performance changes during testing.

Table S1. Summary of resolution and aspect ratio of elastomer surface structures.

Methods	Resolution (nm)	Aspect ratio	Ref.
Stimulus-induced growth	40000	0.05	(45)
	400000	0.625	(46)
	750	0.25	(47)
	11000	0.72	(48)
	380000	0.97	(49)
	100000	0.0155	(55)
	101000	0.97	(56)
	2000	2	(57)
Imprint molding	10000	0.04	(34)
	20000	0.71	(33)
	100000	1	(32)
	300000	0.67	(58)
	1000000	1	(59)
	240000	0.83	(60)
Electron beam direct writing	2000	0.07	(50)
	2000	0.0025	
	1000	0.095	
	500	0.28	
	250	0.24	
	100	0.6	
	100	0.05	

REFERENCES

1. Y. Peng, C. M. Serfass, A. Kawazoe, Y. Shao, K. Gutierrez, C. N. Hill, V. J. Santos, Y. Visell, L. C. Hsiao, Elastohydrodynamic friction of robotic and human fingers on soft micropatterned substrates. *Nat. Mater.* **20**, 1707–1711 (2021).

2. S. Chen, L. Sun, X. Zhou, Y. Guo, J. Song, S. Qian, Z. Liu, Q. Guan, E. M. Jeffries, W. Liu, Y. Wang, C. He, Z. You, Mechanically and biologically skin-like elastomers for bio-integrated electronics. *Nat. Commun.* **11**, 1107 (2020).

3. L. Zhang, H. Chen, Y. Guo, Y. Wang, Y. Jiang, D. Zhang, L. Ma, J. Luo, L. Jiang, Micro–nano hierarchical structure enhanced strong wet friction surface inspired by tree frogs. *Adv. Sci.* **7**, 2001125 (2020).

4. A. Chortos, J. Liu, Z. Bao, Pursuing prosthetic electronic skin. *Nat. Mater.* **15**, 937–950 (2016).

5. Y. Ru, M. Liu, Superwetting gels: Wetting principles, applications, and challenges. *ACS Nano* **19**, 7583–7600 (2025).

6. S. Feng, P. Zhu, H. Zheng, H. Zhan, C. Chen, J. Li, L. Wang, X. Yao, Y. Liu, Z. Wang, Three-dimensional capillary ratchet-induced liquid directional steering. *Science* **373**, 1344–1348 (2021).

7. M. Liu, S. Wang, L. Jiang, Nature-inspired superwettability systems. *Nat. Rev. Mater.* **2**, 17036 (2017).

8. D. Hwang, C. Lee, X. Yang, J. M. Pérez-González, J. Finnegan, B. Lee, E. J. Markvicka, R. Long, M. D. Bartlett, Metamaterial adhesives for programmable adhesion through reverse crack propagation. *Nat. Mater.* **22**, 1030–1038 (2023).

9. J. Chai, Y. Ru, Y. Jia, Y. Yang, H. Zhang, L. Chen, T. Zhao, M. Liu, Friction memory ionogels with hysteretic sticky-slippery transition via thermolocking the metastable state. *Adv. Mater.* **37**, e2416250 (2025).

10. J. Kim, S. Kim, T. Yun, J. H. Kim, C. Son, Y. Lee, K. Kim, H. E. Lee, N. Kim, S. Kim, Shape memory polymer surfaces with controllable roughness for multiscale switchable dry adhesion. *Nat. Commun.* **16**, 4954 (2025).

11. M. Wang, C. Li, S. Napolitano, D. Wang, G. Liu, Quantifying and modeling the crystallinity of polymers confined in nanopores. *ACS Macro Lett.* **13**, 908–914 (2024).

12. V. Narasimhan, R. H. Siddique, J. O. Lee, S. Kumar, B. Ndjamen, J. Du, N. Hong, D. Sretavan, H. Choo, Multifunctional biophotonic nanostructures inspired by the longtail glasswing butterfly for medical devices. *Nat. Nanotechnol.* **13**, 512–519 (2018).

13. S. Y. Chou, P. R. Krauss, P. J. Renstrom, Imprint lithography with 25-nanometer resolution. *Science* **272**, 85–87 (1996).

14. D. Huang, J. Wu, C. Chen, X. Fu, A. H. Brozena, Y. Zhang, P. Gu, C. Li, C. Yuan, H. Ge, M. Lu, M. Zhu, L. Hu, Y. Chen, Precision imprinted nanostructural wood. *Adv. Mater.* **31**, e1903270 (2019).

15. S. Liang, C. Yuan, C. Nie, Y. Liu, D. Zhang, W. C. Xu, C. Liu, G. Xu, S. Wu, Photocontrolled reversible solid-fluid transitions of azopolymer nanocomposites for intelligent nanomaterials. *Adv. Mater.* **36**, e2408159 (2024).

16. L. J. Guo, Nanoimprint lithography: Methods and material requirements. *Adv. Mater.* **19**, 495–513 (2007).

17. C. Guo, L. Feng, J. Zhai, G. Wang, Y. Song, L. Jiang, D. Zhu, Large-area fabrication of a nanostructure-induced hydrophobic surface from a hydrophillic polymer. *ChemPhysChem* **5**, 750–753 (2004).

18. B. Wang, M. Sanviti, A. Alegría, S. Napolitano, Molecular mobility of polymers at the melting transition. *ACS Macro Lett.* **12**, 389–394 (2023).

19. H. D. Rowland, W. P. King, J. B. Pethica, G. L. Cross, Molecular confinement accelerates deformation of entangled polymers during squeeze flow. *Science* **322**, 720–724 (2008).

20. H. Schift, Nanoimprint lithography: An old story in modern times? A review. *J. Vac. Sci. Technol. B* **26**, 458–480 (2008).

21. Z. Hao, A. Ghanekarade, N. Zhu, K. Randazzo, D. Kawaguchi, K. Tanaka, X. Wang, D. S. Simmons, R. D. Priestley, B. Zuo, Mobility gradients yield rubbery surfaces on top of polymer glasses. *Nature* **596**, 372–376 (2021).

22. P. Choi, P. F. Fu, L. J. Guo, Siloxane copolymers for nanoimprint lithography. *Adv. Funct. Mater.* **17**, 65–70 (2007).

23. J. L. Shamshina, P. Berton, Ionic liquids as designed, multi-functional plasticizers for biodegradable polymeric materials: A mini-review. *Int. J. Mol. Sci.* **25**, 1720 (2024).

24. E. Thoms, Z. Song, K. Wang, S. Napolitano, Simple model to predict the adsorption rate of polymer melts. *Phys. Rev. Lett.* **132**, 248101 (2024).

25. H. Tian, C. Bi, Z. Li, C. Wang, B. Zuo, Metastable polymer adsorption dictates the dynamical gradients at interfaces. *Macromolecules* **56**, 4346–4353 (2023).

26. B. Yang, F. Cai, S. Huang, H. Yu, Athermal and soft multi-nanopatterning of azopolymers: Phototunable mechanical properties. *Angew. Chem. Int. Ed Engl.* **132**, 4064–4071 (2020).

27. H.-C. Scheer, N. Bogdanski, M. Wissen, S. Möllenbeck, Impact of glass temperature for thermal nanoimprint. *J. Vac. Sci. Technol. B* **25**, 2392–2395 (2007).

28. C. Probst, C. Meichner, K. Kreger, L. Kador, C. Neuber, H. W. Schmidt, Athermal azobenzene-based nanoimprint lithography. *Adv. Mater.* **28**, 2624–2628 (2016).

29. G. Nian, Z. Chen, X. Bao, M. W. M. Tan, Y. Kutsovsky, Z. Suo, Natural rubber with high resistance to crack growth. *Nat. Sustain.* **8**, 692–701 (2025).

30. W. Kuhn, F. Grün, Beziehungen zwischen elastischen Konstanten und Dehnungsdoppelbrechung hochelastischer Stoffe. *Kolloid-Zeitschrift* **101**, 248–271 (1942).

31. Y. Mao, B. Talamini, L. Anand, Rupture of polymers by chain scission. *Extreme Mech. Lett.* **13**, 17–24 (2017).

32. L. Chen, C. Zhao, J. Huang, J. Zhou, M. Liu, Enormous-stiffness-changing polymer networks by glass transition mediated microphase separation. *Nat. Commun.* **13**, 6821 (2022).

33. X. Zhao, L.-M. Peng, Y. Chen, X.-J. Zha, W.-D. Li, L. Bai, K. Ke, R.-Y. Bao, M.-B. Yang, W. Yang, Phase change mediated mechanically transformative dynamic gel for intelligent control of versatile devices. *Mater. Horiz.* **8**, 1230–1241 (2021).

34. H. Meng, P. Xiao, J. Gu, X. Wen, J. Xu, C. Zhao, J. Zhang, T. Chen, Self-healable macro-/microscopic shape memory hydrogels based on supramolecular interactions. *Chem. Commun.* **50**, 12277–12280 (2014).

35. J. Chung, J. W. Chung, R. D. Priestley, S.-Y. Kwak, Confinement-induced change in chain topology of ultrathin polymer fibers. *Macromolecules* **51**, 4229–4237 (2018).

36. C. Wu, L. Li, Unified description of transportation of polymer chains with different topologies through a small cylindrical pore. *Polymer* **54**, 1463–1465 (2013).

37. Y. Jeong, S. Shin, H. Choi, S. Kim, J. Kim, S. Kwon, K.-Y. Kim, S.-H. Lee, Y.-G. Jung, Y. T. Cho, Fabrication of nano-micro hybrid structures by replication and surface treatment of nanowires. *Crystals* **7**, 215 (2017).

38. G. Chen, S. A. Soper, R. L. McCarley, Free-standing, erect ultrahigh-aspect-ratio polymer nanopillar and nanotube ensembles. *Langmuir* **23**, 11777–11781 (2007).

39. M. S. Kim, S. Shin, W. Y. Kim, S. H. Lee, S. R. Park, S. Kim, Y. T. Cho, Formation of multiscale porous surfaces via evaporation-induced aggregation of imprinted nanowires with highly viscous photocurable materials. *Int. J. Precis. Eng. Manuf.* **26**, 217–225 (2025).

40. W. Zhang, H. Wang, A. T. L. Tan, A. Sargur Ranganath, B. Zhang, H. Wang, J. Y. E. Chan, Q. Ruan, H. Liu, S. T. Ha, D. Wang, V. K. Ravikumar, H. Y. Low, J. K. W. Yang, Stiff shape memory polymers for high-resolution reconfigurable nanophotonics. *Nano Lett.* **22**, 8917–8924 (2022).

41. S. H. Kang, B. Pokroy, L. Mahadevan, J. Aizenberg, Control of shape and size of nanopillar assembly by adhesion-mediated elastocapillary Interaction. *ACS Nano* **4**, 6323–6331 (2010).

42. H. Wang, Q. Ruan, H. Wang, S. D. Rezaei, K. T. P. Lim, H. Liu, W. Zhang, J. Trisno, J. Y. E. Chan, J. K. W. Yang, Full color and grayscale painting with 3D printed low-index nanopillars. *Nano Lett.* **21**, 4721–4729 (2021).

43. B. Pokroy, S. H. Kang, L. Mahadevan, J. Aizenberg, Self-organization of a mesoscale bristle into ordered, hierarchical helical assemblies. *Science* **323**, 237–240 (2009).

44. J. Y. E. Chan, Q. Ruan, M. Jiang, H. Wang, H. Wang, W. Zhang, C.-W. Qiu, J. K. W. Yang, High-resolution light field prints by nanoscale 3D printing. *Nat. Commun.* **12**, 3728 (2021).

45. P. Zhu, Q. Song, S. Bhagwat, F. Mayoussi, A. Goralczyk, N. Nekoonam, M. Sanjaya, P. Hou, S. Tisato, F. Kotz-Helmer, D. Helmer, B. E. Rapp, Generation of precision microstructures based on reconfigurable photoresponsive hydrogels for high-resolution polymer replication and microoptics. *Nat. Commun.* **15**, 5673 (2024).

46. L. Xue, X. Xiong, B. P. Krishnan, F. Puza, S. Wang, Y. Zheng, J. Cui, Light-regulated growth from dynamic swollen substrates for making rough surfaces. *Nat. Commun.* **11**, 963 (2020).

47. D. Chen, C. Ni, L. Xie, Y. Li, S. Deng, Q. Zhao, T. Xie, Homeostatic growth of dynamic covalent polymer network toward ultrafast direct soft lithography. *Sci. Adv.* **7**, eabi7360 (2021).

48. Y. Zhu, J. Li, T. Ma, X. Gao, K. Li, X. Ma, X. Jiang, Self-wrinkling-induced mechanically adaptive patterned surface of photocuring coating for abrasion resistance. *Adv. Mater.* **37**, e2414352 (2024).

49. H. F. Chan, R. Zhao, G. A. Parada, H. Meng, K. W. Leong, L. G. Griffith, X. Zhao, Folding artificial mucosa with cell-laden hydrogels guided by mechanics models. *Proc. Natl. Acad. Sci. U.S.A.* **115**, 7503–7508 (2018).

50. M. J. P. Biggs, M. Fernandez, D. Thomas, R. Cooper, M. Palma, J. Liao, T. Fazio, C. Dahlberg, H. Wheadon, A. Pallipurath, A. Pandit, J. Kysar, S. J. Wind, The functional

response of mesenchymal stem cells to electron-beam patterned elastomeric surfaces presenting micrometer to nanoscale heterogeneous rigidity. *Adv. Mater.* **29**, 1702119 (2017).

51. J. De-La-Cuesta, E. González, A. J. Moreno, A. Arbe, J. Colmenero, J. A. Pomposo, Size of elastic single-chain nanoparticles in solution and on surfaces. *Macromolecules* **50**, 6323–6331 (2017).

52. M. Rubinstein, R. H. Colby, in *Polymer Physics* (Oxford Univ. Press, 2003).

53. J. Hwang, D. G. Lee, H. Yeo, J. Rao, Z. Zhu, J. Shin, K. Jeong, S. Kim, H. W. Jung, A. Khan, Proton transfer hydrogels: Versatility and applications. *J. Am. Chem. Soc.* **140**, 6700–6709 (2018).

54. J. Chen, S. Yao, B. Wang, Q. Yu, B. Xue, P. Yin, Polymer films' residual stress attenuation from the supramolecular complexation with ultra-small nanoparticles for high resolution nanoimprint lithography. *Angew. Chem. Int. Ed Engl.* **64**, e202416759 (2025).

55. S. Zeng, Y. Liu, S. Li, K. Shen, Z. Hou, A. P. Chooi, A. T. Smith, Z. Chen, L. Sun, Smart laser-writable micropatterns with multiscale photo/moisture reconstructible structure. *Adv. Funct. Mater.* **31**, 2009481 (2021).

56. J. Zou, S. Wu, J. Chen, X. Lei, Q. Li, H. Yu, S. Tang, D. Ye, Highly efficient and environmentally friendly fabrication of robust, programmable, and biocompatible anisotropic, all-cellulose, wrinkle-patterned hydrogels for cell alignment. *Adv. Mater.* **31**, e1904762 (2019).

57. J. Kim, J. Yoon, R. C. Hayward, Dynamic display of biomolecular patterns through an elastic creasing instability of stimuli-responsive hydrogels. *Nat. Mater.* **9**, 159–164 (2010).

58. Z. Zhao, C. Li, Z. Dong, Y. Yang, L. Zhang, S. Zhuo, X. Zhou, Y. Xu, L. Jiang, M. Liu, Adaptive superamphiphilic organohydrogels with reconfigurable surface topography for programming unidirectional liquid transport. *Adv. Funct. Mater.* **29**, 1807858 (2019).

59. Z. Zhao, S. Zhuo, R. Fang, L. Zhang, X. Zhou, Y. Xu, J. Zhang, Z. Dong, L. Jiang, M. Liu, Dual-programmable shape-morphing and self-healing organohydrogels through orthogonal supramolecular heteronetworks. *Adv. Mater.* **30**, e1804435 (2018).

60. K. Gong, L. Hou, P. Wu, Hydrogen-bonding affords sustainable plastics with ultrahigh robustness and water-assisted arbitrarily shape engineering. *Adv. Mater.* **34**, e2201065 (2022).

61. P. Ditsche-Kuru, E. S. Schneider, J.-E. Melskotte, M. Brede, A. Leder, W. Barthlott, Superhydrophobic surfaces of the water bug Notonecta glauca: A model for friction reduction and air retention. *Beilstein J. Nanotechnol.* **2**, 137–144 (2011).

62. A. F. Pomerantz, R. H. Siddique, E. I. Cash, Y. Kishi, C. Pinna, K. Hammar, D. Gomez, M. Elias, N. H. Patel, Developmental, cellular and biochemical basis of transparency in clearwing butterflies. *J. Exp. Biol.* **224**, jeb237917 (2021).

63. K. Xu, P. Zi, X. Ding, Learning from biological attachment devices: Applications of bioinspired reversible adhesive methods in robotics. *Front. Mech. Eng.* **17**, 43 (2022).
